# Targeting *Cbx3*/HP1γ Induces LEF-1 and IL-21R to Promote Tumor-Infiltrating CD8 T-Cell Persistence

**DOI:** 10.3389/fimmu.2021.738958

**Published:** 2021-10-06

**Authors:** Phuong T. Le, Ngoc Ha, Ngan K. Tran, Andrew G. Newman, Katharine M. Esselen, John L. Dalrymple, Eva M. Schmelz, Avinash Bhandoola, Hai-Hui Xue, Prim B. Singh, To-Ha Thai

**Affiliations:** ^1^ Department of Pathology, Beth Israel Deaconess Medical Center, Harvard Medical School, Boston, MA, United States; ^2^ Institute of Cell and Neurobiology, Charité-Universitätsmedizin Berlin, Corporate Member of Freie Universität Berlin, Humboldt-Universität zu Berlin and Berlin Institute of Health, Berlin, Germany; ^3^ Division of Gynecologic Oncology, Beth Israel Deaconess Medical Center, Harvard Medical School, Boston, MA, United States; ^4^ Department of Human Nutrition, Food, and Exercise, Virginia Tech, Blacksburg, VA, United States; ^5^ Center for Cancer Research, National Cancer Institute, Bethesda, MD, United States; ^6^ Center for Discovery and Innovation, Hackensack University Medical Center, Nutley, NJ, United States; ^7^ Nazarbayev University School of Medicine, Nur-Sultan, Kazakhstan; ^8^ Cancer Research Institute, Beth Israel Deaconess Medical Center, Harvard Medical School, Boston, MA, United States

**Keywords:** *Cbx3*/HP1γ, LEF-1, IL-21 receptor, CD8+ T-cell persistence, ovarian cancer, melanoma

## Abstract

Immune checkpoint blockade (ICB) relieves CD8^+^ T-cell exhaustion in most mutated tumors, and TCF-1 is implicated in converting progenitor exhausted cells to functional effector cells. However, identifying mechanisms that can prevent functional senescence and potentiate CD8^+^ T-cell persistence for ICB non-responsive and resistant tumors remains elusive. We demonstrate that targeting *Cbx3*/HP1γ in CD8^+^ T cells augments transcription initiation and chromatin remodeling leading to increased transcriptional activity at *Lef1* and *Il21r*. LEF-1 and IL-21R are necessary for *Cbx3*/HP1γ-deficient CD8^+^ effector T cells to persist and control ovarian cancer, melanoma, and neuroblastoma in preclinical models. The enhanced persistence of *Cbx3*/HP1γ-deficient CD8^+^ T cells facilitates remodeling of the tumor chemokine/receptor landscape ensuring their optimal invasion at the expense of CD4^+^ Tregs. Thus, CD8^+^ T cells heightened effector function consequent to *Cbx3*/HP1γ deficiency may be distinct from functional reactivation by ICB, implicating *Cbx3*/HP1γ as a viable cancer T-cell-based therapy target for ICB resistant, non-responsive solid tumors.

## Introduction

Under persistent antigen exposure as in cancer or chronic infections, CD8^+^ effector T cells enter an altered differentiation program known as T-cell exhaustion (T_EX_) (1). In the tumor microenvironment (TME), CD8^+^ progenitor T_EX_ cells express unique transcription factors as well as those shared with naïve and memory cells including the high mobility group (HMG) transcription factor, T-cell factor 1 (TCF-1, encoded by *Tcf7*). They have minimal effector capacity but can be reinvigorated by ICB to proliferate and differentiate into terminally exhausted cells having reduced proliferative capacity but efficient tumor killing ability mediated by perforin 1 (PRF1), granzyme B (GrB) and interferon γ (INF-γ) (2–4). TCF-1 is implicated in the conversion of CD8^+^ progenitor T_EX_ to terminally differentiated T_EX_ (5–9). However, given that only ~20% of all solid tumors and primarily those harboring high mutation loads respond to ICB, in addition to the emergence of ICB resistance (10), there is a need to identify novel targets. In humans and mice, TCF-1, and the related lymphoid enhancer-binding factor 1 (LEF-1) are functionally linked during T-cell development in the thymus and the generation of precursor as well as functional memory CD8^+^ and CD4^+^ T cells (11–19). Nevertheless, a role for LEF-1 in tumor response has not been documented despite *Lef1* expression being detected in human and mouse stem-like tumor infiltrating lymphocyte (TIL) subsets that also express *Tcf7* (7, 9, 20–22).

IL-21R is widely expressed on various innate and adaptive immune cell-lineages including activated CD8^+^ T, CD4^+^ T_FH_ and NK cells. During a chronic viral infection or under IL-2-deprived conditions IL-21R signaling is critical for preventing CD8^+^ T-cell exhaustion (23, 24). In acute viral infections, IL-21R signaling is essential for the proliferation and survival of activated CD8^+^ T cells as well as the generation of long-lived memory cells (25–27). However, the function of IL-21R signaling in cancer is controversial and not completely understood (28–32).

Members of the mammalian heterochromatin protein 1 (HP1) family *Cbx5*/HP1α, *Cbx1*/HP1β and *Cbx3*/HP1γ are small modular proteins around 25kD consisting of an N-terminal chromodomain (CD) and a sequence related C-terminal sequence called the chromo-shadow domain (CSD) that are connected by an unstructured linker region (LR) (33). They are key epigenetic regulators that recognize and bind (“read”) the H3K9me2/3 determinant of the histone code (34). *Cbx5*/HP1α and *Cbx1*/HP1β are usually associated with constitutive heterochromatin while *Cbx3*/HP1γ has a more euchromatic distribution (35, 36). Outside of cytologically-visible heterochromatin, HP1 proteins and H3K9me2/3 assemble into heterochromatin-like domains/complexes having diverse chromatin template-dependent functions (37). HP1 proteins are involved in gene repression/activation, transcriptional elongation, RNA splicing and DNA repair (38–44). *Cbx3*/HP1γ likely controls gene activation through two intimately related processes: transcriptional elongation and co-transcriptional splicing of nascent RNA transcripts (39–41, 45). For example, in the immune system, *Cbx3*/HP1γ is found associated with the transcription elongation complex containing RNA polymerase II (Pol II) within the coding region of the actively transcribed IL-2 gene in stimulated primary T cells (40). By contrast, a role for gene repression is indicated during B-cell development where *Cbx3*/HP1γ is associated with the silenced κ allele implicating *Cbx3*/HP1γ in allelic exclusion (46).

Here, we demonstrate that targeting *Cbx3*/HP1γ promotes increased/sustained expression of LEF-1 and IL-21R in CD8^+^ effector T cells. *Cbx3*/HP1γ deficiency leads to augmented transcription initiation and chromatin remodeling that results in increased transcriptional activity at *Lef1* and *Il21r*. Genetic ablation of *Lef1* and *Il21r* in *Cbx3*/HP1γ-deficient mice causes a loss of tumor CD8^+^ effector T cells accompanied by the reduction of *Ifng*, *Gzmb* and *Prf1* expression, which results in uncontrolled ovarian, melanoma and neuroblastoma growth. Our data establish that LEF-1 and IL-21R are necessary for *Cbx3*/HP1γ-deficient CD8^+^ T cells to maintain their effector capacity and persist in tumors. Our findings together with those of others (22, 47, 48) illustrate the complex mechanisms governing CD8^+^ T-cell effector differentiation and function within a given TME. They underscore the need for continuing exploration of novel targets that can reverse CD8^+^ T-cell dysfunction in ICB resistant and unresponsive solid tumors.

## Methods

### Mice

*Cbx3*/HP1γ floxed mice were generated and provided by Dr. Prim B. Singh (49). These mice were backcrossed to C57BL/6 for twelve generations; they bred and developed normally before and after Cre-mediated deletion. The *Cbx3*/HP1γ transgenic line was generated by inserting a 1 kb fragment of mouse *Cbx3*/HP1γ cDNA, derived from activated CD8^+^ T cells, into EcoR1/Sma1 cloning sites of the VA-hCD2 vector provided by Dr. Dimitris Kioussis (50). The purified *Cbx3*/HP1γ-VA-hCD2 DNA construct was injected into C57BL/6 pronuclei by Dr. Lina Du at the fee-based Dana-Farber/Harvard Cancer Center (DF/HCC) Transgenic Mouse Core. Two founder transgenic lines were produced. The *Lef1* floxed mice were generated as described and provided by Dr. Hai-Hui Xue (12). The following mouse lines were purchased from The Jackson Laboratory: *Il21r* knock out (B6.129-*Il21r^tm1Kopf^*/J) (51), CD8α-Cre transgenic (C57BL/6-Tg(Cd8a-cre)1Itan/J) (52), ROSA26 (B6;129-*Gt(ROSA)26Sor^tm2Sho^*/J) (53), *Prf1* knock out (C57BL/6-*Prf1^tm1Sdz^*/J) (54), and *Ifng* knock out (B6.129S7-*Ifng^tm1Ts^*/J) (55). The B6.SJL line (B6.SJL-*Ptprc^a^*/BoyAiTac) was purchased from Taconic.

### Tumor Cell Lines

The mouse syngeneic ID8 ovarian tumor line was obtained from Dr. Katherine F. Roby under an MTA executed by the University of Kansas Medical Center (56). Dr. Eva M. Schmelz provided the mouse syngeneic MOSE-L_TIC_ *_v_* ovarian tumor line (57, 58). The NB9464 neuroblastoma cell line was a kind gift from Dr. Crystal L. MacKall (Stanford University) (59). The B16-F10 melanoma cell line was purchased from ATCC and tested negative for mycoplasma.

### Antibodies

All flow cytometry fluorochrome-conjugated antibodies were purchased from BioLegend, eBio-sciences or BD Biosciences. The following Western blot and ChIP-tested antibodies were purchased from Cell Signaling: tri-methyl-histone H3 (Lys9) (D4W1U) rabbit mAb #13969, phospho-Rpb1 CTD (Ser2) (E1Z3G) rabbit mAb #13499, phospho-Rpb1 CTD (Ser5) (D9N5I) rabbit mAb #13523, LEF-1 (C12A5) rabbit mAb #2230, cleaved caspase-3 (Asp175) antibody #9661 and phospho-HP1γ (Ser83) Ab #2600. The Western blot and ChIP-tested mouse anti-HP1γ mAb (clone 42s2) was purchased from Millipore Sigma. The rabbit polyclonal to TBR2/Eomes (ab23345) was purchased from Abcam.

### Tumor Induction

Ovarian tumor. Mice were injected intraperitoneally (IP) with ID8 (5 x 10^6^/mouse in 200 µl PBS) or MOSE-L_TICv_ (1 x 10^4^/mouse in 200 µl PBS). On day 120 (ID8) or 36 (MOSE-L_TICv_), when abdominal distension was visible, mice were euthanized, ascites were collected, and volume measured using syringes fitted with 18-gauge needles.

Melanoma. Mice were implanted subcutaneously with B16 tumor cells (1 x 10^5^/mouse in 100µL PBS). Using a digital caliber, B16 tumor size was measured and calculated on day 14 and at 2-day or 3-day intervals until day 20 depending on the size of the tumor, at which time mice were euthanized for analysis.

Neuroblastoma. Mice were implanted subcutaneously with NB-9464 tumor cells (1 x 10^6^/mouse in 100µL PBS). Using a digital caliber, NB-9464 tumor volume was measured and calculated (W x L x 0.4) starting on day 22 and at 2-day intervals until day 30 depending on the size of the tumor, at which time mice were euthanized for analysis.

### Flow Cytometry

Flow cytometry was performed on the Beckman Coulter CytoFLEX LX. Analysis was performed with FlowJo software version 10.8.0 (Tree Star, Inc.). Tumors and ascites were harvested, minced (NBL and melanoma), and cell-suspensions were filtered through 70μm cell-strainers (Fisherbrand). Cells were stained for appropriate surface markers as indicated in figures. Intracellular FOXP3 staining was performed according to manufacturer’s protocol (Biolegend). Briefly, tumor cells (1 x 10^6^) were harvested and washed 2X with staining buffer [PBS, 2% fetal bovine serum (FBS)]. Standard surface staining for intratumoral CD4^+^CD25^+^ T cells was performed. Cells were washed and incubated in fixation/permeabilization buffer (BioLegend) for 30 minutes at room temperature (RT) or 18 hrs at 4°CC. Following incubation, cells were washed 2X with permeabilization buffer (BioLegend) and stained for FOXP3 using anti-mouse FOXP3-PE (BioLegend). Cells were incubated for 30 minutes at RT. Cells were washed 2X with permeabilization buffer and resuspended in FACS buffer for analysis. All antibodies for flow cytometry were purchased from BioLegend: Il-21R-PE/Cy5 (clone 4A9); B220-Pacific Blue (clone RA3-6B2); CD8-PB; CD8-APC/Cy7; CD8-PE/Cy7; CD8-APC (all CD8 antibodies were from clone 53-6.7); CD25-APC/Cy7; CD25-PE (clone PC61 and clone 3C7); NKG2D-PE (clone CX5); CD4-PE; CD4-APC; CD4-FITC (all CD4 antibodies were from clone GK1.5); CD62L-APC (clone MEL-14); CD44-PB; CD44-APC/Cy7 (all CD44 antibodies were from clone IM7); TIM3-APC (clone B8.2C12); Streptavidin-PE; NK1.1-APC (clone PK136); Gr1-APC/Cy7 (clone RB6-8C5); Mac-1-PE (clone M1/70); CXCR5-Biotin (L138D7); FOXP3-PE (MF-14); CD45.2-PE/Cy7 (clone 104).

### *In Vitro* Activation/Differentiation of CD8^+^ T Cells

CD8^+^CD44^–^ T cells were purified from spleen and peripheral lymph nodes using the MojoSort™ Mouse CD8 Naïve T-Cell Isolation Kit (BioLegend, #480044) followed by CD25-depletion using the mouse CD25 MicroBead Kit (Miltenyl Biotec, #130-091-072), all according to manufacturers’ protocols. Naïve CD8^+^ T cells (1 × 10^6^/mL) were activated with plate-bound anti-CD3 (clone 145-2C11, 0.25 μg/ml, BioLegend) and anti-CD28 (clone 37.51, 0.5 μg/ml, BioLegend) in T-cell medium (high glucose DMEM, 10% FBS, penicillin/streptomycin, non-essential amino acids, HEPES, L-glutamate and sodium pyruvate) at 37°C in 10% CO_2_ for 2 days. Cells were then removed from CD3/CD28 activation and re-cultured (5 × 10^5^/ml) in differentiation medium (T-cell medium supplemented with 10 IU/ml rhIL-2 from NCI) at 37°C in 10% CO_2_. Cells were then sub-cultured every day in differentiation medium at 5 × 10^5^/ml each sub-culture. On day 5, cells were harvested and used for Western blots, ChIP-qPCR, qPCR or adoptive T-cell transfer.

### Adoptive T-Cell Transfer

*In vitro*-generated donor CD8^+^ effector T cells (CD45.2^+^) were prepared as above. On day 0, B6.SJL (CD45.1^+^) mice were implanted subcutaneously with NB-9464 (1 x 10^6^/mouse in 100μL PBS) or B16 (1 x 10^5^/mouse in 100μL PBS) tumor cells. On day 10 (B16) or 14 (NB-9464) after tumor induction, mice were treated with donor CD8^+^ effector T cells (3 x 10^6^/mouse in 100μL PBS) *via* tail vein intravenous injection. B16 tumor size and NB-9464 tumor volume were measured and calculated on indicated dates.

### Co-Culturing Effector Cells With B16 Tumor Cells

Target B16 tumor cells were plated in 12-well plates at a density of 5 x 10^5^ cells per well in 750μl of medium (high glucose DMEM, 10% FBS, penicillin/streptomycin, non-essential amino acids, HEPES, L-glutamate and sodium pyruvate). CD8^+^ or CD4^+^ effector T cells were added. For ratio of 1:1 (effector:target), 5 x 10^5^ CD8^+^ or CD4^+^ T cells: 5 x 10^5^ B16 cells were co-cultured in the same well; ratio 5:1, 2.5 x 10^6^ CD8^+^ or CD4^+^ T cells: 5 x 10^5^ B16 cells. Plates were incubated at 37°CC in 5% CO_2_ for 24 hours. Wells were washed to remove non-adherent T cells. Adherent B16 cells were collected and washed with 1 ml cold PBS. Pellets were resuspended in Radio-Immunoprecipitation Assay (RIPA) lysis buffer containing a protease inhibitor cocktail (Roche) and used immediately or stored at –80°CC for further analysis.

### Western Blots

Cells (1 x 10^6^) were lysed with RIPA buffer (Boston BioProducts) containing a protease inhibitor cocktail on ice for 30 minutes. Lysates were centrifuged at 13,000 rpm for 10 minutes at 4°CC. Protein extracts were denatured at 95°CC for 10 minutes, separated by SDS-PAGE, and transferred to PVDF membranes (EMD Millipore). Membranes were probes with primary antibodies. Proteins of interest were detected with HRP-conjugated secondary antibodies and the Pierce™ ECL Western Blotting Substrate (Thermo Fisher Scientific). Membranes were exposed with HyBlot CL Autoradiography films (Denville Scientific, Inc.), and developed with the Kodax X-OMAT 2000 Processor.

### Chromatin Immunoprecipitation Followed by qPCR (ChIP-qPCR) and ChIP Followed by Deep Sequencing (ChIP-Seq)

ChIP was performed using the SimpleChIP Kit #9003 according to manufacturer’s protocol (Cell Signaling). Briefly, 2 x 10^7^ CD8^+^ T cells were used for each ChIP. Cells were fixed in 1% formaldehyde to cross-link proteins to DNA, then lysed with 500μL of lysis buffer containing 1X ChIP buffer and protease inhibitor cocktail. Chromatin was sheared using a Sonic Dismembrator (Fisher Scientific Model 120) at 120W-20kHz power, at 15 seconds per cycle, 45 seconds break in between, for 3 cycles total. Chromatin was subjected to immunoprecipitation using specific antibodies at 4°C overnight with rotation. Following incubation, ChIP grade protein G magnetic beads were added to each ChIP and incubated for 2 hours at 4°C with rotation. Samples were placed in a magnetic separation rack for 2 minutes each time and washed 3X with high and low salt buffers. Chromatin was eluted from antibody/protein G magnetic beads using 1X ChIP elution buffer at 65°C for 30 minutes. Eluted chromatin was collected and subjected to cross-link reversal using 5M NaCl and 20 mg/ml Proteinase K, incubated for 2 hours at 65°C. After reversal of protein-DNA cross-link, the DNA was purified using DNA purification spin columns and eluted with DNA elution buffer provided in the kit. Purified ChIP DNA was immediately used for qPCR. BioRad hard-shell PCR Plates (BioRad) were used. In each well 2μL of purified ChIP DNA was added in triplicates along with 18μl primer mixture, which consisted of 1μL forward primer (5μM), 1μL reverse primer (5μM), 6μL nuclease-free water, and 10μL of 2X QuaniNova SYBR Green PCR Master Mix (Qiagen) or SimpleChIP Universal qPCR Master Mix (Cell Signaling). The plate was centrifuged at 300 RCF for 1 minute and read in the BioRad CFX384 Real-Time System (BioRad). The following qPCR settings were used: initial denaturation 95°C for 3:00 minutes, denatured at 95°C for 0:15 minutes, annealing and extension at 60°C for 1:00 minute, GOTO step denature and extension for a total of 40 cycles. Quantitative PCR result was analyzed, and the IP efficiency was calculated per formula provided in Simple ChIP Kit #9003 (Cell Signaling). Primers are listed in **Table S2**.

ChIP-Seq detailed experiments and analyses have been described previously (60).

### Reverse Transcription-Quantitative Polymerase Chain Reaction (RT-qPCR)

Total RNA was extracted from tumors or activated/differentiated CD8^+^ T cells using TRIzol (Thermo Fisher Scientific). After DNAse I treatment and RNA cleanup with the RNeasy kit (Qiagen), 1 μg of total RNA was reversed transcribed into cDNA using the Invitrogen SuperScript II reverse transcriptase kit according to manufacturer’s recommendations (Thermo Fisher Scientific). The BioRad hard-shell PCR Plates (BioRad) were used. Typically, 5-10 ng of cDNA was added to each well in triplicates along with 7.5μL of the primer mixture, which consisted of 0.5μL forward primer (10μM), 0.5μL reverse primer (10μM), 1.7μL PCR grade water, and 5μL of Roches LightCycler 480 SYBR Green I Master (Roche). The plate was centrifuged at 300 RCF for 1 minute and read in the BioRad CFX384 Real-Time System (BioRad). The qPCR settings: 95°C for 10:00 minutes, 95°C for 0:10 minutes, 62°C for 0:20 minutes, 72°C for 0:20 minutes (Read Plate), GOTO step 2 for 39 times, 92°C for 0:05 minutes, melt curve 65°C to 97°C, at increment of 0.5°C for 1:00 minute (Read Plate), 40°C for 0:10 minutes. Primers are listed in **Table S3**.

### Immunohistochemistry (IHC)

Tumor section processing and TUNEL staining were performed by HistoWiz (Brooklyn, NY). Briefly, all IHC staining is automated using BOND Rx. Slides were treated with Dewax, a solvent-based solution (Leica Biosystems). Tissue sections were fixed by adding 10% neutral buffered formalin for 15 minutes. Slides were treated with Proteinase K at 1:500 dilution. Tissue sections were re-fixed using 10% neutral buffered formalin. Tissue sections were then treated with the equilibration buffer and incubated for 12 minutes at room temperature (RT). Subsequently the TdT reaction mix was added and incubated for 60 minutes at 37°C. The TUNEL reaction was stopped by adding 2X SSC. Tissue slides were treated with Peroxide Block (3-4% Hydrogen Peroxide) followed by wash buffer. Streptavidin HRP was added and incubated for 30 minutes at RT. DAB was added onto the slides and incubated for 10 minutes. Counter-staining was done by adding hematoxylin. Slides were covered using the Sakura Tissue Tek strainer and cover slips then scanned at 40x using the Leica AT2 scanner.

### Statistical Analysis

Statistical analysis of tumor growth was determined by the Graphpad Two-Way Anova, for survival the Log-rank (Mantel-Cox) test and for all others the Graphpad unpaired student t test (comparison between 2 groups) or One-Way Anova (multiple comparisons of group means) were performed. The number of mice used were determined using the GraphPad StatMate^®^, no animals were excluded from the analysis, and no randomization or blinding was applied.

## Results

### *Cbx3*/HP1γ Deficiency Modulates LEF-1 and IL-21R Expression in CD8^+^ Effector T Cells

**Figure 1:**
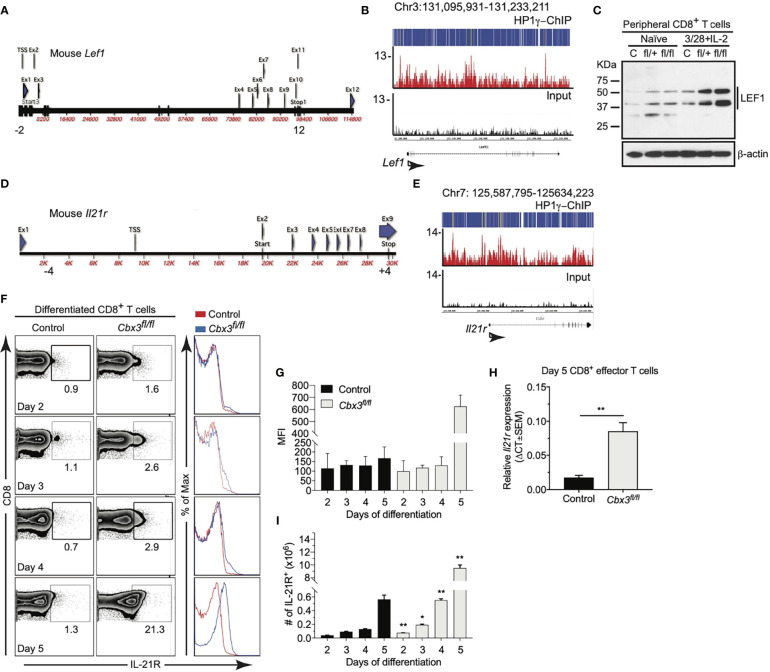
*Cbx3*/HP1γ deficiency modulates LEF-1 and IL-21R expression in CD8^+^ effector T cells. **(A)** Mouse *Lef1* locus. -2 and 12: positions of first and last primer pairs, respectively. **(B)** Read-density tracks of *Cbx3*/HP1γ peaks across *Lef1* identified by ChIP-Seq using chromatin from wt day 5 activated/differentiated CD8^+^ T cells (pooled from 3 mice); y axis: number of reads per million mapped per 25-bp window; representative of 2 independent ChIP-Seq runs. **(C)** LEF-1 expression was evaluated by Western immunoblot using total protein lysates from activated/differentiated control and *Cbx3*/HP1γ-deficient CD8^+^ T cells; 3/28+IL2: CD8^+^ T cells activated/differentiated for 5 days with plate-bound anti-CD3 and anti-CD28 plus IL-2; C: control (CD8α-Cre or wt); fl/+: *Cbx3^fl/+^* and fl/fl: *Cbx3^fl/fl^* (CD8^+^ T-cell-restricted deletion of *Cbx3/*HP1γ using the CD8α-Cre mouse); n = 3; representative of 3 experiments. **(D)** Mouse *Il21r* locus. -4 and 4: positions of first and last pair of primers, respectively. **(E)** Read-density tracks of *Cbx3*/HP1γ peaks across *Il21r* were determined by ChIP-Seq as in **(B)**. **(F)** IL-21R expression levels on activated/differentiated CD8^+^ T cells by flow; numbers: percent cells; representative of 3 experiments; n = 3. **(G)** IL-21R mean fluorescence intensity (MFI) was evaluated from **(F)**. **(H)** Relative expression of *Il21r* in day 5 activated/differentiated CD8^+^ T cells was assessed by RT-qPCR and normalized to *Gapdh*; Graphpad unpaired student t-test: **p ≤ 0.01; representative of 3 experiments. **(I)** Number of CD8^+^IL-21R^+^ T cells in cultures was calculated from **(F)**; Graphpad unpaired student t-test: *p ≤ 0.05, **p ≤ 0.01.

Previously we showed that germline deletion of *Cbx3*/HP1γ impairs lymphoid-tissue germinal center (GC) reaction and high-affinity antibody response against a thymus (T)-dependent antigen (Ag) in a CD8^+^ T-cell-intrinsic manner (61). *Cbx3*/HP1γ deficiency releases the effector capacity of CD8^+^ T cells to control neuroblastoma (NBL) growth (60). However, it is not understood why *Cbx3*/HP1γ-deficient CD8^+^ effector T cells are not subjected to functional senescence and persist in NBL or if they can control tumors with varied mutation loads. To ensure *Cbx3*/HP1γ deletion was restricted to CD8^+^ T cells, all experiments were performed using the CD8α-Cre strain (52) crossed with our *Cbx3*/HP1γ-floxed mice (resulting animals were *Cbx3*/HP1γ^fl/+^ and *Cbx3*/HP1γ^fl/fl^; collectively designated as *Cbx3*/HP1γ-deficient). Cre recombinase from this CD8α-Cre strain was active in CD8^+^CD4^–^ T cells not in CD4^+^CD8^–^ or CD11b^+^ cells (**Figure S1A**). Ablation of *Cbx3*/HP1γ in CD8^+^ T cells resulted in a near or complete loss of protein expression and phosphorylation (**Figure S1B**). Expression of *Bcl6* and *Tbx21* (T-bet) (62) mRNAs was increased in *Cbx3*/HP1γ-deficient CD8^+^ effector T cells over control cells (**Figure S1C**). By contrast *Eomes* transcript and protein levels were reduced (**Figures S1C, D**). We observed an upregulation of *Prf1*, *Gzmb* and *Ifng* mRNA expression in *Cbx3*/HP1γ-deficient CD8^+^ effector T cells compared to control cells (**Figure S1E**). More cleaved caspase 3 (CC3) was detected in B16 melanoma tumor cells co-cultured with *Cbx3*/HP1γ-deficient CD8^+^ effector T cells compared to control co-cultures (**Figure S1F**). *Cbx3*/HP1γ deficiency in CD4^+^ T cells did not enhance tumor cells killing. Thus, after activation, *Cbx3*/HP1γ-deficient CD8^+^ T cells differentiate into effector-like cells armed with a heightened effector/killing capacity to induce tumor-cell apoptosis *in vitro*. To determine mechanisms conferring persistence on *Cbx3*/HP1γ-deficient CD8^+^ effector T cells, ChIP-Seq data was analyzed (60). In wild-type day 5 activated/differentiated CD8^+^ T cells, *Cbx3*/HP1γ was bound to the 5’ untranslated region (UTR) surrounding transcriptional start sites (TSSs) of *Lef1* (**Figures 1A, B**) and *Il21r* (**Figures 1D, E**) (**Table S1**). Western immunoblots showed an induction of LEF-1 expression in activated/differentiated control CD8^+^ T cells; however, its expression was further increased when *Cbx3*/HP1γ was ablated (**Figure 1C**). LEF-1 levels in control and *Cbx3*/HP1γ-deficient thymocytes were similar (**Figure S1G**). The effects of IL-21 on IL-21R expression are controversial. In humans, IL-21 was shown to induce IL-21R expression on naïve CD8^+^ T cells (63). In mice, IL-21 exposure led to the downregulation of its receptor expression on all T-cell subsets (64). To mitigate IL-21 confounding effects, all experiments were done without added IL-21. In the absence of exogenous IL-21, activated/differentiated *Cbx3*/HP1γ-deficient CD8^+^ T cells expressed more IL-21R protein (measured as mean fluorescence intensity or MFI) and transcript, compared to control cells (**Figures 1F–H**). There was a steady expansion of *Cbx3*/HP1γ-deficient CD8^+^IL-21R^+^ T cells after activation/differentiation while the growth of control cells remained low throughout the activation/differentiation period (**Figures 1F, I**). IL-21R was not detected on thymocytes from control or *Cbx3*/HP1γ-deficient mice (**Figure S1H**). We establish that *Cbx3*/HP1γ deficiency induces the sustained increase of LEF-1 and IL-21R in CD8^+^ effector T cells as well as enhancing their effector capacity. IL-21R elevated expression likely provides signals mediating *Cbx3*/HP1γ-deficient CD8^+^ effector T-cell expansion in the absence of exogenous IL-21.

### *Cbx3*/HP1γ Regulates *Lef1* and *Il21r* Transcription Initiation and Chromatin Remodeling

**Figure 2:**
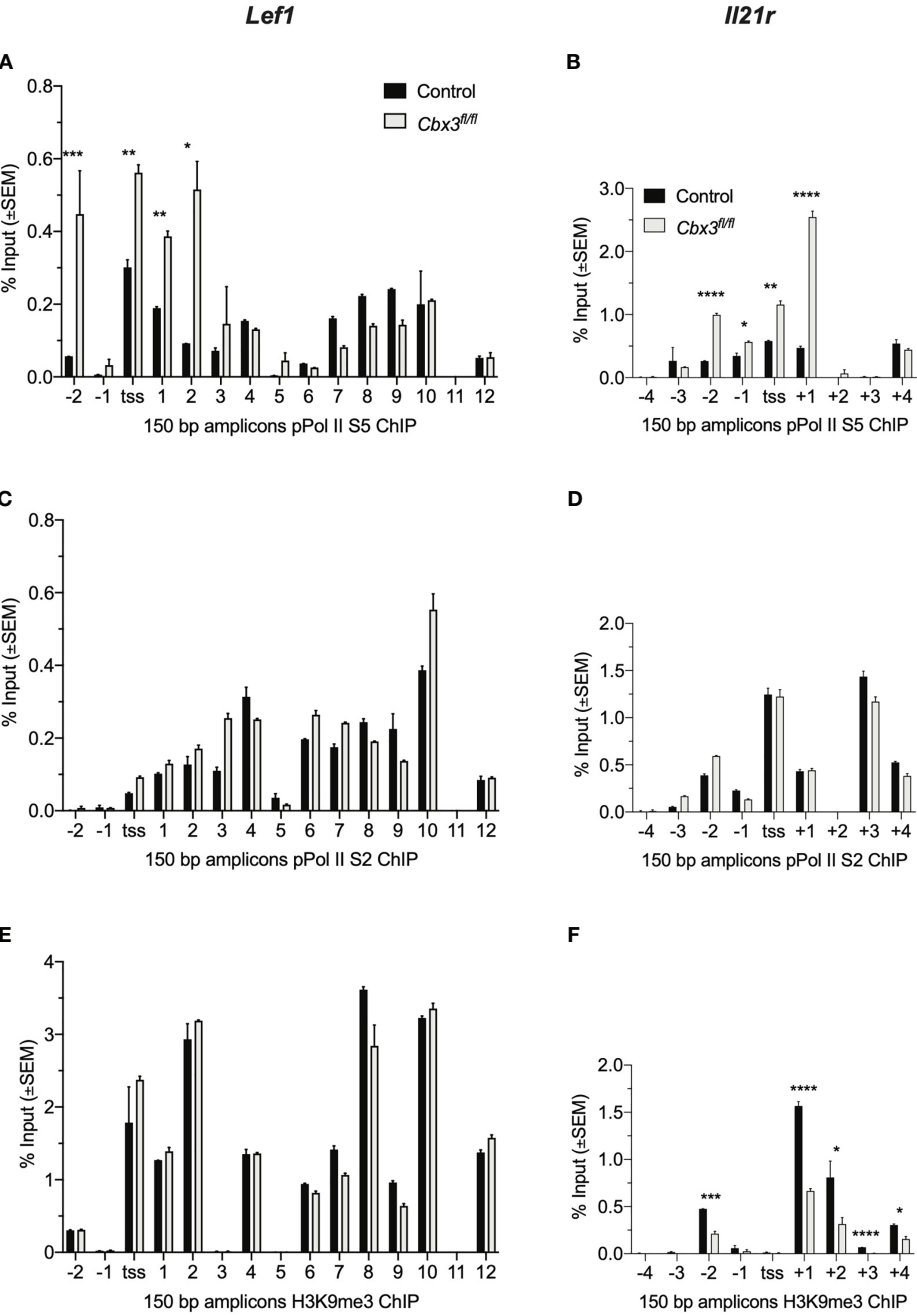
*Cbx3*/HP1γ regulates *Lef1* and *Il21r* transcription initiation and chromatin remodeling. **(A, B)** Levels of Pol II S5 bound to *Lef1* **(A)** and *Il21r* **(B)** were quantified by ChIP-qPCR using chromatin from day 5 activated/differentiated CD8^+^ T cells. TSS: transcription start site; numbers on X axis: positions of primers along the two loci; 150 bp products amplified by *Lef1* or *Il21r* primers. **(C, D)** Levels of Pol II S2 bound to *Lef1* **(C)** and *Il21r* **(D)** were quantified as in **(A, B)**. **(E, F)** H3K9me3 deposition on *Lef1* **(E)** and *Il21r* **(F)** was quantified as in **(A, B)**. Graphpad unpaired student t-test: *p ≤ 0.05, **p ≤ 0.01, ***p ≤ 0.001, ****p ≤ 0.0001; representative of 4 independent ChIPs using pooled chromatin from 3 mice.

The rate of gene expression is governed in part by RNA Pol II initiation, elongation and/or chromatin remodeling. Pol II is phosphorylated at serine 5 (Pol II S5) during initiation of transcription while phosphorylation at serine 2 (Pol II S2) generally indicates chromatin remodeling concomitant with transcriptional elongation. To test whether alterations in transcriptional initiation, elongation and/or chromatin remodeling caused *Lef1* and *Il21r* increased expression in *Cbx3*/HP1γ-deficient CD8^+^ effector T cells, ChIP-qPCR experiments were performed using ChIP-tested antibodies specific for Pol II S2 or S5, and H3K9me3. These results revealed augmented levels of Pol II S5 in or around TSSs of *Lef1* and *Il21r* loci in *Cbx3*/HP1γ-deficient CD8^+^ effector T cells compared to control cells (**Figures 2A, B**). Similar levels of Pol II S2 density were observed in control and *Cbx3*/HP1γ-deficient CD8^+^ T cells (**Figures 2C, D**). The levels of H3K9me3 deposition at *Lef1* locus was unaltered (**Figure 2E**). However, there was a general loss of H3K9me3 around the TSS of *Il21r* locus in *Cbx3*/HP1γ-deficient CD8^+^ effector T cells (**Figure 2F**). Thus, genetic deletion of *Cbx3*/HP1γ in CD8^+^ effector T cells results in enhanced transcription initiation at *Lef1* and *Il21r* loci, with chromatin remodeling activity taking place in an extended region around the TSS of the *Il21r* locus. These changes likely underpinned the increased and sustained transcriptional activity at *Lef1* and *Il21r*.

### *Cbx3*/HP1γ-Deficient CD8^+^ Effector T Cells Persist and Cause Tumor Rejection

**Figure 3:**
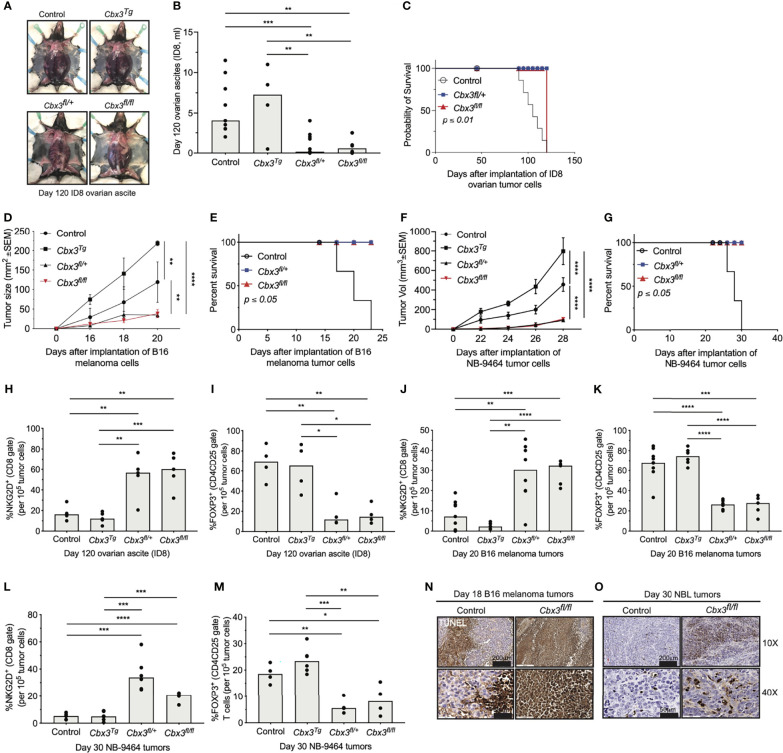
*Cbx3*/HP1γ-deficient CD8^+^ effector T cells can persist and cause tumor rejection. **(A)** Ascites from control (CD8α-Cre or wt), *Cbx3^Tg^*, *Cbx3^fl/+^* and *Cbx3^fl/fl^* mice (X axis) were visualized on day 120 after IP injection of mouse ID8 ovarian tumor cells; *Cbx3^Tg^*: *Cbx3/*HP1γ T-cell-restricted expression driven by the human *Cd2* promoter; *Cbx3^fl/+^* and *Cbx3^fl/fl^*: CD8^+^ T-cell-restricted deletion of *Cbx3/*HP1γ using the CD8α-Cre mouse. **(B)** Ovarian ascites of mice in **(A)** were measured; bars: group median; Graphpad unpaired student t-test: **p≤0.01, ***p≤0.001; each symbol = one mouse; n = 5-8; representative of 2 experiments. **(C)** Survival curves of ovarian tumor-bearing mice were determined; Graphpad log-rank (Mantel-Cox) test; n = 5-8. **(D)** B16 tumor cells were injected subcutaneously (sc) and growth was assessed starting on day 14 after tumor-cell injection then every 2 days through day 20; Graphpad two-way ANOVA: **p ≤ 0.01, ****p ≤ 0.0001; n = 5; representative of 3 experiments. **(E)** Survival curves of B16 melanoma tumor-bearing mice; Graphpad log-rank (Mantel-Cox) test; n = 5. **(F)** NB-9464 tumor cells were injected sc, NBL tumor volume was determined every 2 days starting on day 22 through day 28; Graphpad two-way ANOVA: ****p ≤ 0.0001; n = 5; representative of 2 experiments. **(G)** Survival curves of NBL tumor-bearing mice; Graphpad log-rank (Mantel-Cox) test, n = 5. **(H, J, L)** Frequencies of CD8^+^NKG2D^+^ T cells in ovarian ascites, B16 and NBL tumors from control (CD8α-Cre or wt), *Cbx3^Tg^*, *Cbx3^fl/+^* and *Cbx3^fl/fl^* mice; data were extracted from flow analysis (**Figure S2C, E, G**); bars: group median; Graphpad unpaired student t-test: **p ≤ 0.01, ***p ≤ 0.001, ****p ≤ 0.0001; each symbol = one mouse; n = 5-8; representative of 2-3 experiments. **(I, K, M)** Frequencies of CD4^+^FOXP3^+^ Tregs in ovarian ascites, B16 and NBL tumors (**Figure S2D, F, H**); bars: group median; Graphpad unpaired student t-test: *p ≤ 0.05, **p ≤ 0.01, ***p ≤ 0.001, ****p ≤ 0.0001; each symbol = one mouse; n = 5-8; representative of 2-3 experiments. **(N, O)** Apoptotic cells (brown) in B16 melanoma and NBL tumor sections were identified by TUNEL staining; representative of 4 tumors from each mouse strain.

**Figure 4:**
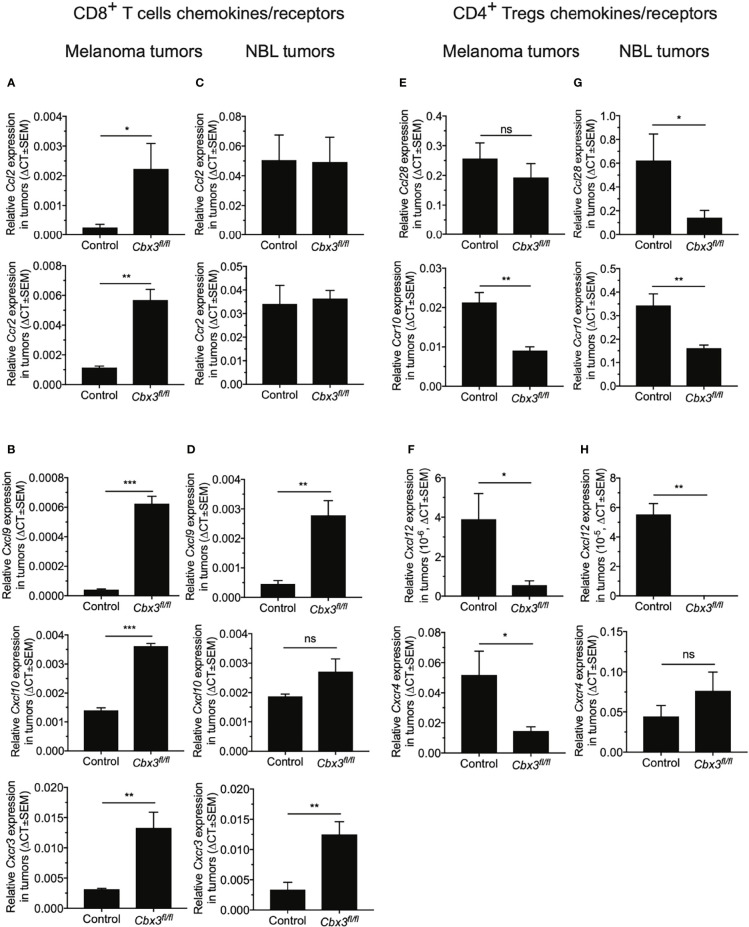
*Cbx3*/HP1γ-deficient CD8^+^ T cells remodel the tumor chemokine/receptor landscape. **(A, B)** Relative expression of chemokines and receptors mediating CD8^+^ T cells trafficking into B16 tumors was determined by RT-qPCR and normalized to *Gapdh*. **(C, D)** Relative expression of chemokines and receptors mediating CD8^+^ T cells trafficking into NBL tumors was evaluated by RT-qPCR and normalized to *Gapdh*. **(E, F)** Relative expression of chemokines and receptors inducing CD4^+^ Tregs homing into tumors was normalized to *Gapdh*. **(G, H)** Relative expression of chemokines and receptors inducing CD4^+^ Tregs homing into NBL tumors was assessed. X axis: mouse strains; Graphpad unpaired student t-test: *p ≤ 0.05, **p ≤ 0.01, ***p ≤ 0.001; ns, not significant; representative of 4 experiments; n = 4.

The increased/sustained expression of LEF-1 and IL-21R together with the enhanced effector capacity exhibited by *Cbx3*/HP1γ-deficient CD8^+^ effector T cells suggest they can persist to control tumor development. Thus, the ability of *Cbx3*/HP1γ-deficient CD8^+^ T cells to eradicate solid tumors was evaluated using the mouse ID8 or MOSE-L_TICv_ ovarian, B16 melanoma and NB-9464 NBL tumor models. Ovarian and NBL tumors have low mutation rates, no clear defining tumor-associated antigens (TAAs) and are minimally responsive to ICB (65–67). Accordingly, syngeneic ID8 or MOSE-L_TIC_ *_v_* ovarian tumor cells were injected intraperitoneally (IP) into control and *Cbx3*/HP1γ-deficient mice. Mice were monitored until abdominal distension was visible, which indicated increased ascites and mirrored metastasis observed in humans. Tumor growth and production of ascites were inhibited in *Cbx3*/HP1γ-deficient mice compared to controls or mice ectopically expressing *Cbx3*/HP1γ driven by the human T-cell-restricted *Cd2* promoter (*Cbx3*/HP1γ^Tg^) (**Figures 3A, B** and **S2A, B**). As a result, *Cbx3*/HP1γ-deficient mice lived longer than controls (**Figure 3C**). *Cbx3*/HP1γ-deficient mice were equally effective in reducing B16 melanoma (**Figure 3D**) and NB-9464 NBL tumor burden (**Figure 3F**), leading to their increased survival compared to controls (**Figures 3E, G**). On day 120 after ID8 injection, there was an enrichment of CD8^+^NKG2D^+^ effector T cells and a decrease in CD4^+^CD25^+^FOXP3^+^ regulatory T cells (Tregs) in ascites from *Cbx3*/HP1γ-deficient mice compared to control or *Cbx3*/HP1γ^Tg^ animals (**Figures 3H, I** and **S2C, D**). Similarly, enrichment of CD8^+^NKG2D^+^ effector T cells and decrease in CD4^+^CD25^+^FOXP3^+^ Tregs were observed in B16 melanoma as well as NBL tumors (**Figures 3J–M** and **S2E–H**). Comparable frequencies of NK1.1^+^NKG2D^+^ or CD4^+^NKG2D^+^ T cells were recovered from tumors of control, *Cbx3*/HP1γ^Tg^ and *Cbx3*/HP1γ-deficient mice (**Figure S3**). Expression of *Ifng*, *Gzmb* and *Prf1* was elevated in B16 melanoma and NBL tumors excised from *Cbx3*/HP1γ-deficient mice compared to controls or *Cbx3*/HP1γ^Tg^ animals (**Figures S4A–F**). TUNEL staining revealed more apoptotic tumor cells (brown) in melanoma and NBL tumors from *Cbx3*/HP1γ-deficient mice compared to controls (**Figures 3N, O**). Genetic ablation of *Prf1* in *Cbx3*/HP1γ-deficient animals led to uncontrolled B16 melanoma growth and decreased TUNEL positivity indicative of reduced tumor-cell apoptosis (**Figures 4SG, H**) while NBL tumor burden was incompletely inhibited and tumor-cell death was readily detected (**Figures S4I, J**). By contrast, *Ifng* deletion resulted in uncontrolled NBL tumor growth in *Cbx3*/HP1γ-deficient mice (**Figure S4K**). Thus, PRF1 and INF-γ likely mediate the killing of tumors in *Cbx3*/HP1γ-deficient mice. To show that *Cbx3*/HP1γ-deficient CD8^+^ effector T cells cause tumor rejection *in vivo*, CD8^+^ T cells (CD45.2^+^) were activated/differentiated *in vitro* (see *Methods*). On day 5 after activation/differentiation, CD8^+^ T cells were collected for adoptive T-cell therapy in tumor bearing congenic B6.SJL (CD45.1^+^) recipients (**Figure S5A**). Treatment with *Cbx3*/HP1γ-deficient CD8^+^ effector T cells alone resulted in a statistically significant decrease in B16 melanoma and NBL tumor burden compared to treatment with control cells (**Figures S5B, C**). Within B16 melanoma tumors, there was an enrichment of transferred *Cbx3*/HP1γ-deficient CD8^+^NKG2D^+^ effector T cells, not transferred control or endogenous CD8^+^ effector T cells (**Figures S5D, E**). Possible contaminating CD45.2^+^CD4^+^NKG2D^+^ and CD45.2^+^NK1.1^+^NKG2D^+^ T cells were not detected in tumors indicating that they were not the source of tumor killing (**Figures S5F–I**). These results suggested a role for NKG2D in tumor control by *Cbx3*/HP1γ-deficient CD8^+^ effector T cells. To test this hypothesis, B16 and NB-9464 tumor cells were injected subcutaneously into control and *Cbx3*/HP1γ-deficient mice. On day 2, a subset of tumor-bearing *Cbx3*/HP1γ-deficient mice were treated with the blocking/non-depleting anti-mouse NKG2D antibody HMG2D, and thereafter once per week. NKG2D blockade resulted in uncontrolled tumor growth and decreased survival of treated animals (**Figures S6A–D**), accompanied by the reduction of *Cbx3*/HP1γ-deficient CD8^+^NKG2D^+^ effector T cells in tumors, to a level similar to PBS-treated control mice (**Figures S6E, F**). NKG2D blockade did not affect CD4^+^NKG2D^+^ and NK1.1^+^NKG2D^+^ T-cell frequencies (**Figures S6G, H**). Together, our results demonstrate that *Cbx3*/HP1γ-deficient CD8^+^ effector T cells expressing NKG2D can persist and cause tumor rejection, irrespective of tumor mutation status. The engagement of NKG2D with its ligands expressed on tumor cells likely facilitates tumor killing by *Cbx3*/HP1γ-deficient CD8^+^ effector T cells through the enhanced production of PRF1, GrB and INF-γ. Additionally, B16 and NBL tumors have differential sensitivity to killing by PRF1 and IFN-γ, and surveying for their presence in the TME alone may not reveal the precise mechanism whereby tumor cells are eradicated. Furthermore, deletion of even one *Cbx3*/HP1γ allele is sufficient to control solid tumor growth suggesting that *Cbx3*/HP1γ is haploinsufficient.

### *Cbx3*/HP1γ-Deficient CD8^+^ T Cells Remodel the Tumor Chemokine/Receptor Landscape

CD8^+^ effector T cells homing to tumors is mediated primarily by the interaction between CCL2/CCR2 and CXCL9/CXCL10/CXCR3 chemokine/receptor pairs while CD4^+^ Tregs trafficking to tumors is induced mostly by CCL28/CCR10 and CXCL12/CXCR4 (68–77). Chemokines are generally produced by tumor-associated myeloid cells including dendritic cells (DCs) and macrophages (MØs) whereas their cognate receptors are expressed on T cells as well as other immune cells. It has been proposed that changes in the tumor chemokine/receptor landscape influence anti-tumor responses. Whether CD8^+^ effector T cells residing in the TME participate in remodeling the tumor chemokine landscape is not clearly established. RT-qPCR assays demonstrated that *Ccl2*/*Ccr2* and *Cxcl9/Cxcl10*/*Cxcr3* levels were higher in B16 melanoma tumors from *Cbx3*/HP1γ-deficient mice than those from controls (**Figures 4A, B**). In NBL tumors from *Cbx3*/HP1γ-deficient mice, *Ccl2*/*Ccr2* and *Cxl10* remained similar to those of controls whereas *Cxcl9*/*Cxcr3* levels were elevated (**Figures 4C, D**). These results indicate that homing of *Cbx3*/HP1γ-deficient CD8^+^ effector T cells to B16 melanoma tumors is likely mediated by CCL2/CCR2 and CXCL9/CXCL10/CXCR3 interactions. *Cbx3*/HP1γ-deficient CD8^+^ effector T cells likely depend on CXCL9/CXCR3 engagement to invade NBL tumors. Compared to controls, *Ccr10* and *Cxcl12*/*Cxcr4* levels were decreased in B16 melanoma tumors from *Cbx3*/HP1γ-deficient mice (**Figures 4E, F**). In NBL tumors from *Cbx3*/HP1γ-deficient mice, *Ccl28/Ccr10* and *Cxcl12* expression was reduced (**Figures 4G, H**). Thus, CCR10 and CXCL12/CXCR4 may regulate homing of CD4^+^ Tregs to B16 melanoma tumors while invasion of NBL tumors by CD4^+^ Tregs is likely governed by CCL28/CCR10 and CXCL12. Decreased levels of CD4^+^ Treg-attracting chemokine/receptor pairs may be causing the diminished presence of these cells in the respective tumors. In short, the presence of *Cbx3*/HP1γ-deficient CD8^+^ effector T cells in the TME induces remodeling of the chemokine/receptor landscape that favors their optimal trafficking into tumors at the expense of CD4^+^ Tregs.

### LEF-1 and IL-21R Are Indispensable for Halting Tumor Growth

**Figure 5:**
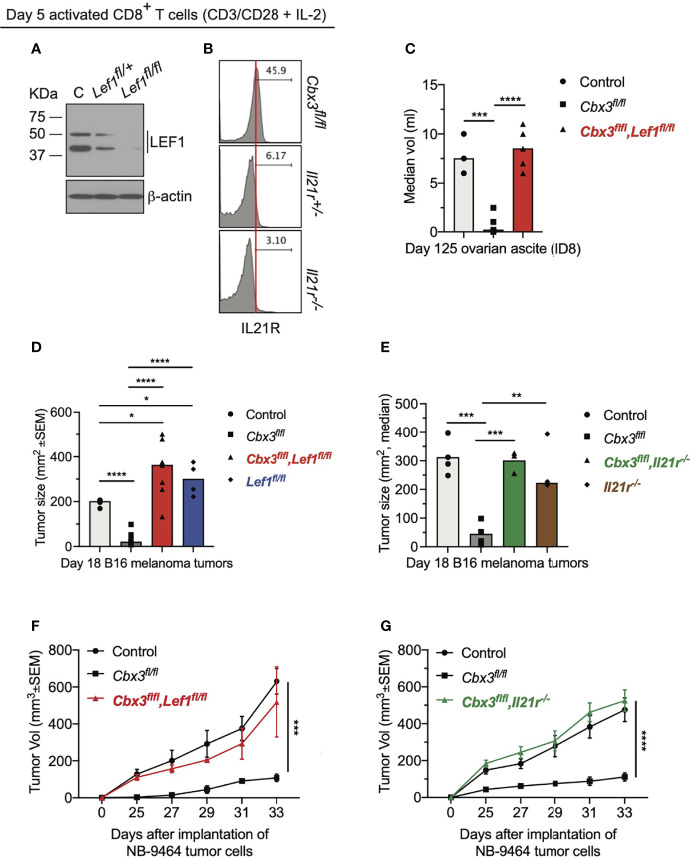
LEF-1 and IL-21R are indispensable for halting tumor growth. **(A)** Western blot of LEF-1 expression was done using total protein lysates of day 5 activated/differentiated CD8^+^ T cells; C: *Cbx3*/HP1γ-deficient; *Lef1^fl/+^* and *Lef1^fl/fl^*: *Cbx3*-*Lef1*-deficient; representative of 4 experiments. **(B)** IL-21R expression was measured by flow; *Cbx3^fl/fl^*: *Cbx3*/HP1γ-deficient; *Il21r^+/-^* and *Il21r^-/-^*: *Cbx3*-*Il21r*-deficient; representative of 4 experiments. **(C)** Ovarian ascites were measured on day 125 after tumor injection; bars: group median; Graphpad unpaired student t-test: ***p ≤ 0.001, ****p ≤ 0.0001; each symbol = one mouse; n = 3-5; *Cbx3^fl/fl^Lef1^fl/fl^*: *Cbx3*-*Lef1*-deficient; representative of 2 experiments. **(D)** B16 melanoma tumor burden was determined on day 18 after tumor injection; bars: group median; Graphpad unpaired student t-test: *p ≤ 0.05, ****p ≤ 0.0001; each symbol = mouse; for each genotype n = 4-7; *Lef1^fl/fl^*: *Lef1* ablation alone using CD8α-Cre mice; representative of 3 experiments. **(E)** On day 10 after injection of tumor cells, tumor bearing B6SJ/L mice (CD45.1^+^) were treated with *in vitro*-activated/differentiated CD8^+^ T cells (CD45.2^+^) from control, *Cbx3^fl/fl^*, *Cbx3^fl/fl^*,*Il21r^-/-^* (*Cbx3*-*Il21r*-deficient) or *Il-21r^-/-^* (germline deleted) mice; **p ≤ 0.01, ***p ≤ 0.001; n = 3-4 recipients; representative of 2 experiments. **(F)** NBL tumor burden was determined starting on day 25 after injection of tumor cells then every 2 days until day 33; Graphpad two-way ANOVA: ***p≤0.001; n = 2-5; representative of 2 experiments. **(G)** On day 14 after NBL injection, tumor bearing B6SJ/L mice were treated with *in vitro*-activated/differentiated CD8^+^ T cells from control, *Cbx3^fl/fl^* or *Cbx3^fl/fl^*,*Il21r^-/-^* (*Cbx3*-*Il21r*-deficient) mice; ****p ≤ 0.0001, n = 3-4 recipients; representative of 2 experiments.

To establish that LEF-1 and IL-21R contribute to the control of tumor growth by *Cbx3*/HP1γ-deficient CD8^+^ effector T cells, compound mutant mice were created (**Figure S7A**). In the *Cbx3*-*Lef1*-deficient mouse, *Lef1* and *Cbx3*/HP1γ deletion was restricted to CD8^+^ T cells; in the *Cbx3*-*Il21r*-deficient mouse, *Cbx3*/HP1γ ablation was restricted to CD8^+^ T cells while *Il21r* was deleted in all tissues. LEF-1 and IL-21R expression was abolished in deficient CD8^+^ T cells compared to control cells (**Figures 5A, B**). Loss of one *Lef1* or *Il21r* allele showed a decrease in protein levels that were almost completely abrogated upon deletion of both alleles. Progenitor T-cell development proceeded normally in the thymus of compound mutant animals (**Figures S7B, C**). Mesenteric lymph nodes (mLNs) of compound mutant mice displayed normal frequencies of mature CD8^+^ and CD4^+^ T cells (**Figures S7D, E**). Normal ratios of naïve (CD44^–^CD62L^+^), effector (CD44^+^CD62L^–^) and memory (CD44^+^CD62L^+^) T cells were observed in the mLNs of compound mutant animals (**Figures S7F, G**). In the mLNs, IL-21R expression was restricted to the CD8^+^ effector (CD44^+^CD62L^–^) T-cell population and was also dependent on gene dosage (**Figure S7H**). IL-21R was not detected on any CD4^+^ T-cell populations (**Figure S7I**). To determine LEF-1 contribution to tumor control, ID8 (ovarian), B16 (melanoma) or NB-9464 (NBL) tumor cells were injected into *Lef1*-deficient, *Cbx3*-*Lef1*-deficient and control mice. CD8^+^ T-cell-restricted *Lef1* ablation resulted in uncontrolled ovarian, melanoma and NBL tumor growth (**Figures 5C, D, F**). Tumor burden in *Lef1*- and *Cbx3*-*Lef1*-deficient animals was higher than that observed for *Cbx3*/HP1γ-deficient and control mice. Because *Il21r* was deleted in all tissues, adoptive transfer of activated/differentiated CD8^+^ T cells from *Il21r* ^-/-^, *Cbx3*-*Il21r*-deficient and control animals was performed. Melanoma and NBL growth proceeded unchecked in tumor-bearing B6.SJL congenic mice treated with control, *Il21r* ^-/-^ or *Cbx3*-*Il21r*-deficient CD8^+^ effector T cells compared to animals receiving *Cbx3*/HP1γ-deficient CD8^+^ effector T cells (**Figures 5E, G**). Our data establish that LEF-1 and IL-21R are necessary for the optimal control of ovarian, melanoma and NBL tumor growth. Moreover, LEF-1 expression in control CD8^+^ effector T cells is induced, yet they are less capable of persisting in tumors, suggesting there may be a threshold of expression level required for LEF-1 to function effectively. IL-21R expression is confined to the CD8^+^ T-cell effector population and its loss following deletion suggest that IL-21R function is essential to this population, which is critical for halting tumor development.

### LEF-1 and IL-21R Are Required for CD8^+^ Effector T Cells to Persist in Tumors

**Figure 6:**
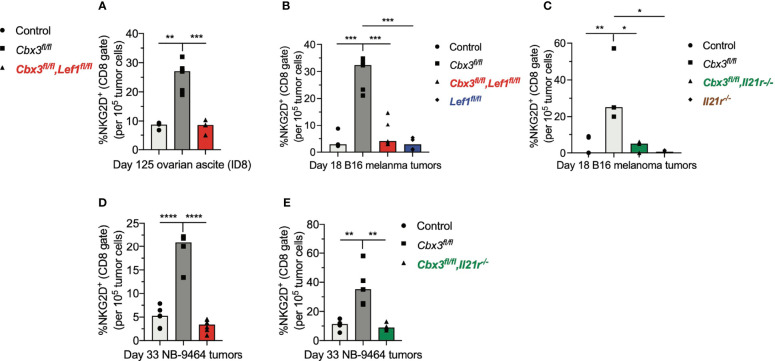
LEF-1 and IL-21R are required for CD8^+^ effector T cells to persist in tumors. **(A)** Frequencies of CD8^+^NKG2D^+^ T cells in ovarian ascites (from **Figure 5**) were extracted from flow analysis; bars: group median; Graphpad unpaired student t-test: **p ≤ 0.01, ***p ≤ 0.001; each symbol = one mouse. **(B, C)** Frequencies of CD8^+^NKG2D^+^ T cells in B16 melanoma tumors (from **Figure 5**) were extracted from flow analysis; bars: group median; Graphpad unpaired student t-test: *p ≤ 0.05, **p ≤ 0.01, ***p ≤ 0.001; each symbol = one mouse. **(D, E)** Frequencies of CD8^+^NKG2D^+^ T cells in NBL tumors (from **Figure 5**) were extracted from flow analysis; bars: group median; Graphpad unpaired student t-test: **p ≤ 0.01, ****p ≤ 0.0001; each symbol = one mouse.

The inability of *Lef1-*, *Il21r-*, *Cbx3*-*Lef1*- and *Cbx3*-*Il21r*-deficient CD8^+^ T cells to halt tumor development suggests that LEF-1 and IL-21R are necessary for their persistence in tumors. Analyses of tumors from *Cbx*3-*Lef1*-deficient mice revealed that the frequencies of CD8^+^NKG2D^+^ T cells were reduced to control levels in all three tumors (**Figures 6A, B, D**). CD8^+^NKG2D^+^ T cells were also decreased to control levels in B16 tumors from *Lef1*-deficient mice (**Figure 6B**). There was no enrichment of CD8^+^NKG2D^+^ T cells in B16 or NBL tumors from animals treated with *Cbx3*-*Il21r*-deficient CD8^+^ effector T cells (**Figures 6C, E**). Likewise, CD8^+^NKG2D^+^ T cells were not augmented in B16 tumors from mice receiving *Il21r^-/-^* CD8^+^ effector T cells (**Figure 6C**). CD8^+^TIM3^+^CXCR5^+^ (progenitor exhausted) or CD8^+^TIM3^+^CXCR5^-^ (terminally exhausted) T-cell populations were not perturbed (**Figure S8**). The frequency of endogenous NK1.1^+^NKG2D^+^, CD4^+^NKG2D^+^ T and myeloid cells in all tumor types was similar to that of controls (**Figure S9**). These results demonstrate that *Lef1* and/or *Il21r* genetic ablation results in the exclusive loss of CD8^+^ effector T cells in all three tumor types. Our data underscore the fact that LEF-1 and IL-21R are requisite for the persistence of *Cbx3*/HP1γ-deficient CD8^+^ effector T cells in tumors with varied mutation loads that are ICB responsive (B16 melanoma) or non-responsive (ovarian and NBL).

### LEF-1 and IL-21R Are Required to Maintain Effector Activity in Tumors

**Figure 7:**
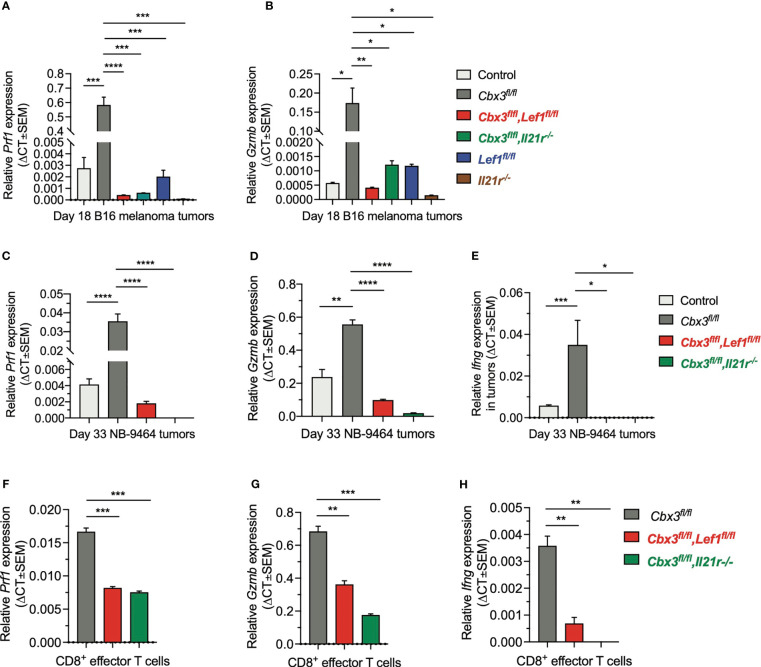
LEF-1 and IL-21R are required for CD8^+^ T cells to maintain their effector capacity. **(A, B)** *Prf*1 and *Gzmb* relative expression in B16 melanoma tumors was determined by RT-qPCR; Graphpad unpaired student t-test: *p ≤ 0.05, **p ≤ 0.01, ***p ≤ 0.001, ****p ≤ 0.0001; n = 3 tumors; representative of 3 experiments. **(C–E)** *Prf1*, *Gzmb* and *Ifng* relative expression in NBL tumors was determined by RT-qPCR; Graphpad unpaired student t-test: *p ≤ 0.05, **p ≤ 0.01, ***p ≤ 0.001, ****p ≤ 0.0001; n = 3 tumors; representative of 3 experiments. **(F–H)** *Prf1*, *Gzmb* and *Ifng* relative expression in day 5 *in vitro*-activated/differentiated CD8^+^ T cells from *Cbx3*/HP1γ-deficient and compound mutant mice was quantified by RT-qPCR; Graphpad unpaired student t-test: **p ≤ 0.01, ***p ≤ 0.001; representative of 3 experiments.

To show that uncontrolled tumor growth resulted primarily from the loss of CD8^+^ T-cell effector capacity, RT-qPCR assays were done using tumor RNA samples from compound mutant and control mice. *Prf1* and *Gzmb* levels in melanoma tumors from control and compound mutant mice as well as those treated with *Cbx3*-*Il21r*- or *Il21r*-deficient CD8^+^ effector T cells were low compared to *Cbx3*/HP1γ-deficient mice (**Figures 7A, B**). *Prf1*, *Gzmb* and *Ifng* expression in NBL tumors from *Cbx*3-*Lef1*-deficient mice and those treated with *Cbx3*-*Il21r*-deficient CD8^+^ effector T cells was diminished compared to *Cbx3*/HP1γ-deficient animals (**Figures 7C–E**). Next, we asked whether the loss of effector capacity is inherent to *Lef1* and/or *Il21r* deficiency or is caused by extrinsic factors in the TME. RT-qPCR analyses showed that *Prf1*, *Gzmb* and *Ifng* levels were reduced in *in vitro*-generated CD8^+^ effector T cells from compound mutant mice compared to those from *Cbx3*/HP1γ-deficient cells (**Figures 7F–H**). Therefore, our findings demonstrate that LEF-1 and IL-21R are indispensable for maintaining *Prf1*, *Gzmb* and *Ifng* in tumors. The loss of CD8^+^ effector T cells likely contributes to the observed blunted effector activity observed in tumors from *Lef1-* and *Cbx3*-*Lef1*-deficient mice as well as those treated with *Il21r-* or *Cbx3*-*Il21r*-deficient CD8^+^ T cells.

## Discussion

We establish that genetic ablation of *Cbx3*/HP1γ induces the elevated, sustained expression of factors conferring both persistence and heightened effector/killing capacity on CD8^+^ T cells, which in turn enables them to control the growth of diverse tumor types. Once in the TME, *Cbx3*/HP1γ-deficient CD8^+^ T cells can remodel the chemokine/receptor landscape to ensure their optimal trafficking into tumors at the expense of CD4^+^ Tregs. Mechanistically, *Cbx3*/HP1γ deficiency allows for a higher rate of transcriptional initiation and chromatin remodeling at *Lef1* and *Il21r* loci. Consequently, *Cbx3*/HP1γ-deficient CD8^+^ T cells express elevated levels of LEF-1 and IL-21R that can mitigate functional senescence and enable their persistence in tumors without the assistance of ICB.

To date, no essential functions for LEF-1 are identified in CD8^+^ effector T cells despite *Lef1* expression being detected in human and mouse stem-like tumor infiltrating lymphocyte (TIL) subsets that also express *Tcf7*. Here, we provide compelling evidence that LEF-1 has a critical and non-redundant function in tumor rejection. The exclusive increase of *Cbx3*/HP1γ-deficient CD8^+^ effector T cells in tumors and their subsequent disappearance when *Lef1* is ablated suggest that LEF-1 is requisite for their persistence in varied tumor types irrespective of mutational status. *Lef1*- and *Cbx3*-*Lef1*-deficient mice, in which *Tcf7* is not deleted, fail to control tumor growth suggesting that *Lef1* function is not redundant. Nevertheless, it is possible that in our models LEF-1^+^CD8^+^ and TCF-1^+^CD8^+^ T cells represent two separate populations with defined functions, and the former subset is endowed with an enhanced proliferative potential, or TCF-1 expression is downregulated in LEF-1^+^CD8^+^ T cells. Our data are distinct to those found with ICB where TCF-1 activity is implicated in the conversion of CD8^+^ T_EX_ progenitor cells into terminally differentiated effector cells that can control the growth of tumors with high mutation rates. Instead, our findings are consistent with those recently published by Weber and colleagues (78). These authors demonstrate that CAR-T cells, when allowed to rest after receptor signaling is shut off, can recover from exhaustion, and regain their anti-tumor activity, in the absence of ICB or other pharmacological interventions. *Lef1* expression is upregulated in rested CAR-T cells not in exhausted CAR-T cells, treated or not treated with anti-PD1. Although TCF-1 expression is similar in exhausted and rested CAR-T cells, the exhausted population cannot kill tumor cells while rested cells can. Moreover, Carr and colleagues report that LEF-1 plays a primary role in the post selection and effector fate differentiation of iNKT2 cells in the thymus (79). Similarly, Reya and colleagues show that LEF-1 is required for progenitor B-cell proliferation and survival through Wnt signaling (80). In the latter two models, the authors posit that LEF-1 exhibits a more dominant role while TCF-1 function is not sufficient for the differentiation and expansion of the immune-cell populations examined.

Our observation that IL-21R^+^CD8^+^ effector T cells increase in number after activation and are lost upon *Il21r* deletion suggest that IL-21R provides survival and expansion signals to these cells. This is in keeping with works showing that during a chronic viral infection or under IL-2-deprived conditions, IL-21R signaling is critical for preventing CD8^+^ T-cell exhaustion (23, 24). In acute viral infections, IL-21R signaling is essential for the proliferation and survival of activated CD8^+^ T cells as well as the generation of long-lived memory cells (25–27). In these models, IL-21R can activate the STAT1/STAT3 signaling pathways, which subsequently upregulate pro-survival factors BCL-2 and BCL-X_L_ and downregulate TRAIL (26, 27). Furthermore, several lines of evidence indicate that IL-21R is involved in tumor immunity. First, high expression of IL-21R on CD8^+^ T cells in tumors correlates positively with overall survival and lack of tumor recurrence in hepatocellular carcinoma (HCC) patients (28). Second, in mice, IL-21R signaling reduces accumulation of myeloid derived suppressor cells (MDSCs) in the TME to control rapid HCC growth and maintains an immunological memory response to tumor re-challenge (28). Third, newly diagnosed HER2^+^ breast cancer patients with higher *Il21r* expression may have a reduced risk of distant relapse when treated with trastuzumab (anti-HER2/ErB2 mAb) in combination with chemotherapy; IL-21R expression on CD8^+^ effector T cells, not NK cells, is required for optimal anti-ErB2 mAb efficacy (29). Moreover, similar to HER2^+^ breast tumors, *Il21* is not readily detected in B16 and NBL tumors from control or *Cbx3*/HP1γ-deficient mice. By contrast, IL-21 is visible in subcutaneous tumors (28). Differences in IL-21 expression are also observed in viral infection models. IL-21 is produced during both the acute and chronic phases; however, its levels decrease during the chronic phase (81, 82). This reduction is attributed to a contraction in the number of virus specific CD4^+^ T cells, which are the primary source of IL-21 production. Though reduced, IL-21 produced during the chronic phase can provide help to CD8^+^ T cells to resolve the infection. Thus, local endogenous IL-21 production in tumors may also be temporal. Because IL-21R expression is induced and sustained in *Cbx3*/HP1γ-deficient CD8^+^ effector T cells without exogenous IL-21, we posit that whether IL-21 production is high or low during tumor development, it can still positively affect *Cbx3*/HP1γ-deficient CD8^+^ T-cell effector functions.

Within tumors, activated tumor-associated-MØs and/or -DCs set up a milieu to be either activating or suppressing through the production of chemokines that attract CD8^+^ T cells or CD4^+^ Tregs (77). Our data demonstrate that *Cbx3*/HP1γ-deficient CD8^+^ effector T cells can remodel the chemokine landscape favoring their accumulation while preventing CD4^+^ Tregs from trafficking into tumors. It is tempting to speculate that the imbalance of chemokine production is caused by functional alterations and/or selective expansion of tumor-associated myeloid cells imposed by *Cbx3*/HP1γ-deficient CD8^+^ effector T cells.

Our results suggest a model whereby *Cbx3*/HP1γ normally restrains the effector and persistence potential of CD8^+^ T cells, which eventually succumb to functional inactivation and apoptosis thus incapable of controlling tumor development. Removal of *Cbx3*/HP1γ restraint allows CD8^+^ T cells to become effector-like cells armed with an intrinsic heightened killing and persistence capacity to control tumor growth; LEF-1 function and IL-21R signaling are necessary. Alternatively, *Cbx3*/HP1γ-deficient CD8^+^ effector T cells are preferentially recruited to tumors compared to control cells. Moreover, our model does not preclude the possibility that LEF-1 and IL-21R may also be implicated in CD8^+^ T-cell responses downstream of ICB. Since mouse and human *Cbx3*/HP1γ peptides are 100% identical, we suspect that human *Cbx3*/HP1γ will behave identically to the mouse ortholog: *Cbx3*/HP1γ deficiency would have the same effects on human CD8^+^ T cells. Ovarian and neuroblastoma are tumors with low mutation rates that respond minimally to ICB, yet their growth is effectively controlled by *Cbx3*/HP1γ-deficient CD8^+^ effector T cells. Thus, our findings provide a rationale for targeting *Cbx3*/HP1γ in human T cells to treat tumors harboring low mutation loads and do not respond to ICB.

## Data Availability Statement

The datasets presented in this study can be found in online repositories. The names of the repository/repositories and accession number(s) can be found below: https://www.ncbi.nlm.nih.gov/geo/GSE183238.

## Ethics Statement

The animal study was reviewed and approved by BIDMC Institutional Animal Care and Use Committee.

## Author Contributions

THT, NH, and PL conceived the project and wrote the original draft with input and guidance from KE, JD, EMS, AB, H-HX, PS, THT, PL, NH, and NT designed and performed experiments. PL, NH, AB, H-HX, PS, and THT analyzed data and wrote the final manuscript. All authors contributed to the article and approved the submitted version.

## Funding

This work was supported by NIH grants AI099012, CA198263, Friends for Life Neuroblastoma Research Program and The Mayer Family Fund.

## Conflict of Interest

The authors declare that the research was conducted in the absence of any commercial or financial relationships that could be construed as a potential conflict of interest.

## Publisher’s Note

All claims expressed in this article are solely those of the authors and do not necessarily represent those of their affiliated organizations, or those of the publisher, the editors and the reviewers. Any product that may be evaluated in this article, or claim that may be made by its manufacturer, is not guaranteed or endorsed by the publisher.

## Supplementary Material

Supplementary Figure 1 Enhanced effector functions of *Cbx3*/HP1γ-deficient CD8+ T cells. **(A)** Cre activity (EGFP+) was restricted to CD8+CD4– spleen T cells from a cross between the reporter mouse ROSA-EGFP and the CD8α-Cre mouse. **(B)** Western immunoblot of total (tHP1γ) and phosphorylated (pHP1γ) *Cbx3*/HP1γ levels were evaluated in day 5 activated/differentiated pooled spleen and peripheral lymph node (pLN) CD8+ T cells using plate-bound anti-CD3/CD28 + IL-2 (10 IU/ml). *Cbx3fl/+ and Cbx3fl/fl*: CD8+ T-cell-restricted deletion of *Cbx3*/HP1γ in mice using the CD8α-Cre strain; collectively designated as *Cbx3*/HP1γ-deficient; representative of 3 experiments; n = 3 mice. **(C)** Relative expression of *Bcl6*, *Eomes* and *Tbx21* was quantified by RT-qPCR using RNA samples of day 5 activated/differentiated CD8+ T cells. Unit 1 indicates no change; n = 3; representative of 3 experiments. **(D)** Western immunoblot showed EOMES expression in same cells as **(B)**. **(E)** Relative expression of *Gzmb*, *Prf1* and *Ifng* was etermined by RT-qPCR using same RNA samples as in **(C)**. **(F)** Cleaved (activated) caspase 3 (CC3) in protein lysate of washed cells was detected 24 hours after coculture using an antibody specific to CC3. CD8:target = effector CD8+ T cells cocultured with target; control: CD8α-Cre; *Cbx3fl/+*: CD8-restricted deletion of *Cbx3*/HP1γ with the CD8α-Cre mouse. CD4:target = day 2 activated CD4+ T cells from control (CD4-Cre) or *Cbx3fl/fl* mice cocultured with target; *Cbx3fl/fl*: CD4-restricted deletion of *Cbx3*/HP1γ with the CD4-Cre mouse; target: B16 melanoma tumor cells. 1:1= 1 effector cell to 1 target cell; 5:1 = 5 effector cells to one target cell; Un: no added effector cells; representative of 3 experiments. **(G)** Western immunoblot showed LEF-1 expression in thymocytes from control and *Cbx3*/HP1γ-deficient mice; representative of 3 experiments. **(H)** Flow histograms showed lack of IL-21R expression on thymocytes from control and *Cbx3*/HP1γdeficient mice; representative of 3 experiments.

Supplementary Figure 2 Reduction of ovarian ascites in *Cbx3*/HP1γ-deficient mice. **(A)** Images showed ovarian ascites in control and *Cbx3*/HP1γ-deficient mice injected (IP) with the syngeneic mouse ID8 cell line; control: CD8α-Cre; *Cbx3fl/+ and Cbx3fl/fl*: *Cbx3*/HP1γ-deficient mice; yellow outlines: tumor nodules (supporting data for **Figures 3A, B**). **(B)** Ovarian ascites in control and *Cbx3*/HP1γ-deficient mice injected (IP) with the syngeneic mouse MOSE-LTICv cell line were quantified and graphed as group median; control: CD8α-Cre; *Cbx3fl/fl*: *Cbx3*/HP1γ-deficient mice; Graphpad student unpaired t-test; *p≤0.05; n = 4-5.; representative of 2 experiments. **(C, E, G)** Flow analysis showed frequencies of CD8+NKG2D+ effector T cells in ovarian ascites, B16 and NBL tumors from control (CD8α-Cre or wt), *Cbx3Tg*, *Cbx3fl/+* and *Cbx3fl/fl* mice; *Cbx3Tg*: T-cell-restricted expression of *Cbx3/*HP1γ driven by the human *Cd2* promoter (supporting data for **Figures 3H, J, L**). **(D, F, H)** Flow analysis depicted the frequencies of CD4+FOXP3+ regulatory T cells (Tregs) in ovarian ascites, B16 and NBL tumors from control (CD8α-Cre or wt), *Cbx3Tg*, *Cbx3fl/+* and *Cbx3fl/fl* mice (supporting data for **Figures 3I, K, M**).

Supplementary Figure 3 No enrichment of CD4+NKG2D+ or NK1.1+NKG2D+ T cells in tumors (supporting data for **Figure 3**). **(A, C, E)** Frequencies of CD4+NKG2D+ T cells in ovarian ascites, B16 and NBL tumors were extracted from flow analysis; bars: group median; Graphpad unpaired student t-test: ns, not significant; each symbol = one mouse; n = 2-8. **(B, D, F)** Frequencies of NK1.1+NKG2D+ T cells in ovarian ascites, B16 and NBL tumors were extracted from flow analysis; bars: group median; each symbol = one mouse; n = 2-8.

Supplementary Figure 4
*Prf1*, *Gzmb* and *Ifng* expression in tumors. **(A–C)** Relative expression of *Ifng*, *Gzmb* and *Prf1* in B16 melanoma tumors were quantified by RT qPCR and normalized to *Gapdh*; Graphpad unpaired student t-test: *p≤0.05, **p≤0.01, n = 4 tumors from each mouse strain; representative of 4 experiments. **(D–F)** Relative expression of *Ifng*, *Gzmb* and *Prf1* in NBL tumors was normalized to *Gapdh*; Graphpad unpaired student t-test: *p≤0.05, **p≤0.01, ***p≤0.001; n = 3 tumors from each mouse strain; representative of 3 experiments. **(G)** B16 melanoma tumor growth in *Cbx3fl/fl*,*Prf1-/-* (*Cbx3*/HP1γ-deficient mice lacking *Prf1*), *Cbx3fl/fl* (*Cbx3*/HP1γ-deficient) and control (CD8α-Cre and wt) mice was measured; Graphpad two-way ANOVA; ****p≤0.0001;n = 6; representative of 2 experiments. **(H)** Apoptotic cells (brown) in B16 melanoma tumors from mice in **(A)** were identified by TUNEL staining; representative of 2 experiments. **(I)** NBL tumor burden in mice implanted with NB-9464 tumor cells was measured; *Cbx3fl/fl*,*Prf1-/-*: *Cbx3*/HP1γ-deficient mice lacking *Prf1*; *Cbx3fl/fl*: *Cbx3*/HP1γ-deficient; control: CD8α-Cre and wt; Graphpad two-way ANOVA; ****p≤0.0001; n = 3-5; representative of 2 experiments. **(J)** Apoptotic cells (brown) in NBL tumors from mice in **(C)** were identified by TUNEL staining; representative of 2 experiments. **(K)** NBL tumor growth in *Cbx3fl/fl*,*Ifng-/-* (*Cbx3*/HP1γ-deficient mice lacking *Ifng*) was evaluated; *Cbx3fl/fl* (*Cbx3*/HP1γ-deficient) and control (CD8α-Cre and wt) mice; Graphpad two-way ANOVA; *p≤0.05, **p≤0.01; n = 3-4; representative of 2 experiments.

Supplementary Figure 5 Direct cause of tumor rejection by *Cbx3*/HP1γ-deficient CD8+ effector T cells. **(A)** Schematic of ACT. **(B)** 10 days after implantation of B16 tumor cells, B6.SJL recipients (CD45.1+) were treated once with exogenous CD8+ effector T cells (CD45.2+); bars: group median; Graphpad unpaired student t-test: *p≤0.05, **p≤0.01; each symbol = one mouse; n = 4-5 recipients; representative of 2 experiments. **(C)** 14 days after implantation of N-9464 tumor cells, B6.SJL recipients were treated once with exogenous CD8+ effector T cells; Graphpad two-way ANOVA: ****p≤0.0001; n = 5 recipients; representative of 2 experiments. **(D)** Frequencies of CD8+ effector T cells in B16 melanoma tumors after ACT; A: B6.SJL mice treated with control CD8+ T cells, B: B6.SJL mice treated with *Cbx3fl/+* CD8+ T cells, C: B6.SJL mice treated with *Cbx3fl/fl* CD8+ T cells; grey bars: transferred CD8+ effector T cells (CD45.2+); black bars: endogenous CD8+ effector T cells (CD45.1+); bars: group median; Graphpad unpaired student t-test: *p≤0.05, **p≤0.01, ns: not significant; each symbol = one mouse; n = 4-5 recipients from B. **(E)** Staining and gating strategies for panel D; gated on total live tumor cells obtained from tumors of B6.SJL recipients from B. **(F, G)** Frequencies of CD4+CD45.2+ T cells in B16 melanoma tumors **(F)** and spleens **(G)** from B6.SJL recipients treated with control (CD8α-Cre) or *Cbx3*/HP1γ-deficient CD8+ effector T cells; data were extracted from flow analysis; bars: group median; each symbol = one mouse; n = 4-5 recipients as in **(D)**. **(H, I)** Frequencies of NK1.1+CD45.2+ T cells in B16 tumors **(H)** and spleens **(I)** from B6.SJL recipients treated with control or *Cbx3*/HP1γ-deficient CD8+ effector T cells; data were extracted from flow analysis; bars: group median; each symbol = one mouse; n = 4-5 recipients from B.

Supplementary Figure 6 Effects of NKG2D blockade on tumor growth. **(A)** B16 melanoma tumor burden was assessed after NKG2D blockade using the anti-mouse NKG2D antibody HMG2D; Graphpad two-way ANOVA: ****p≤0.0001; *Cbx3fl/fl*-NKG2D TX: *Cbx3fl/fl* mice injected with HMG2D one day after injection of tumor cells; control and *Cbx3fl/fl* mice were injected with PBS; n = 5 received Ab, 10 received PBS; representative of 2 experiments. **(B)** Survival curves of B16 tumor-bearing mice after NKG2D blockade. **(C)** NBL tumor burden was evaluated after NKG2D blockade; Graphpad two-way ANOVA: **p≤0.01; n = 4 received Ab, 10 received PBS; representative of 2 experiments. **(D)** Survival curves of NBL tumor-bearing mice after NKG2D blockade. **(E, F)** Flow analysis showed frequencies of CD8+NKG2D+ T cells in B16 melanoma tumors; bars: group median; Graphpad student unpaired t-test: **p≤0.01; each symbol = one mouse; *Cbx3fl/fl*-NKG2D TX: *Cbx3*/HP1γ-deficient mice receiving the anti-mouse NKG2D antibody HMG2D; control and *Cbx3*/HP1γfl/fl: PBS treated. **(G, H)** Frequencies of CD4+NKG2D+ and NK1.1+NKG2D+ T cells in B16 melanoma tumors in **(A)**.

Supplementary Figure 7 Normal T-cell development in the thymus of compound mutant mice. **(A)** Generation of compound mutant mice. **(B, C)** T-cell development in the thymus of *Cbx3*-*Lef1*-deficient and *Cbx3*-*Il21r*-deficient mice was assessed; numbers: percent cells; DN1-DN4: double negative (CD4–CD8–) progenitor T-cell populations gated from bottom left quadrants of top flow plots. **(D, E)** CD4+ and CD8+ ratio was evaluated in mLNs from compound mutant mice; numbers: percent cells. **(F, G)** mLN naïve (CD44–CD62L+), effector (CD44+CD62L–) and memory (CD44+CD62L+) cells in CD4+ as well as CD8+ T-cell compartments of compound mutant mice were assessed; numbers: percent cells. **(H, I)** Flow analysis was done to evaluate IL21R expression on mLN CD8+ **(H)** and CD4+ **(I)** effector (CD44+CD62L–), naïve (CD44–CD62L+) and memory (CD44+CD62L+) T cells from *Cbx3*/HP1γ-deficient and *Cbx3*-*Il21r*-deficient mice; numbers: percent cells. For all panels, n = 3 for each genotype; representative of 3 experiments.

Supplementary Figure 8 Analysis of CD8+ progenitor and terminally exhausted T cells. **(A)** Frequencies of progenitor exhausted (TIM3+CXCR5+) and terminally exhausted (TIM3+CXCR5–) CD8+ T cells in all tumors from *Cbx3fl/flLef1fl/fl* (*Cbx3*-*Lef1*-deficient), *Cbx3fl/fl* (*Cbx3*/HP1γ-deficient) and control (CD8α-Cre or wt) mice were assessed; n = 3-5; representative of 2 experiments. **(B)** Progenitor exhausted (TIM3+CXCR5+) and terminally exhausted (TIM3+CXCR5–) CD8+ T cells were identified in tumors from B6.SJL mice treated with *Cbx3fl/flIl21r-/-*, *Cbx3fl/fl* or control CD8+ effector T cells (CD45.2+); n = 3-5 recipients; representative of 2 experiments.

Supplementary Figure 9 Analysis of tumor infiltrating immune populations. **(A)** Frequencies of NK1.1+NKG2D+, CD4+NKG2D+ T, Gr1+ and Mac1+ myeloid cells in tumors from *Cbx3fl/flLef1fl/fl* (*Cbx3*-*Lef1*-deficient) and control animals; representative of 2 experiments. **(B)** Frequencies of endogenous NK1.1+NKG2D+, CD4+NKG2D+ T, Gr1+ and Mac1+ myeloid cells in tumors from B6.SJL mice treated with *Cbx3fl/flIl21r-/-* and control CD8+ effector T cells; representative of 2 experiments.
