# Supplementary figures and images for: Targeting Cbx3/HP1γ Induces LEF-1 and IL-21R to Promote Tumor-Infiltrating CD8 T-Cell Persistence
Source: Front Immunol. 2021 Oct 6;12:738958. doi: 10.3389/fimmu.2021.738958 (PMC8549513; doi:10.3389/fimmu.2021.738958)

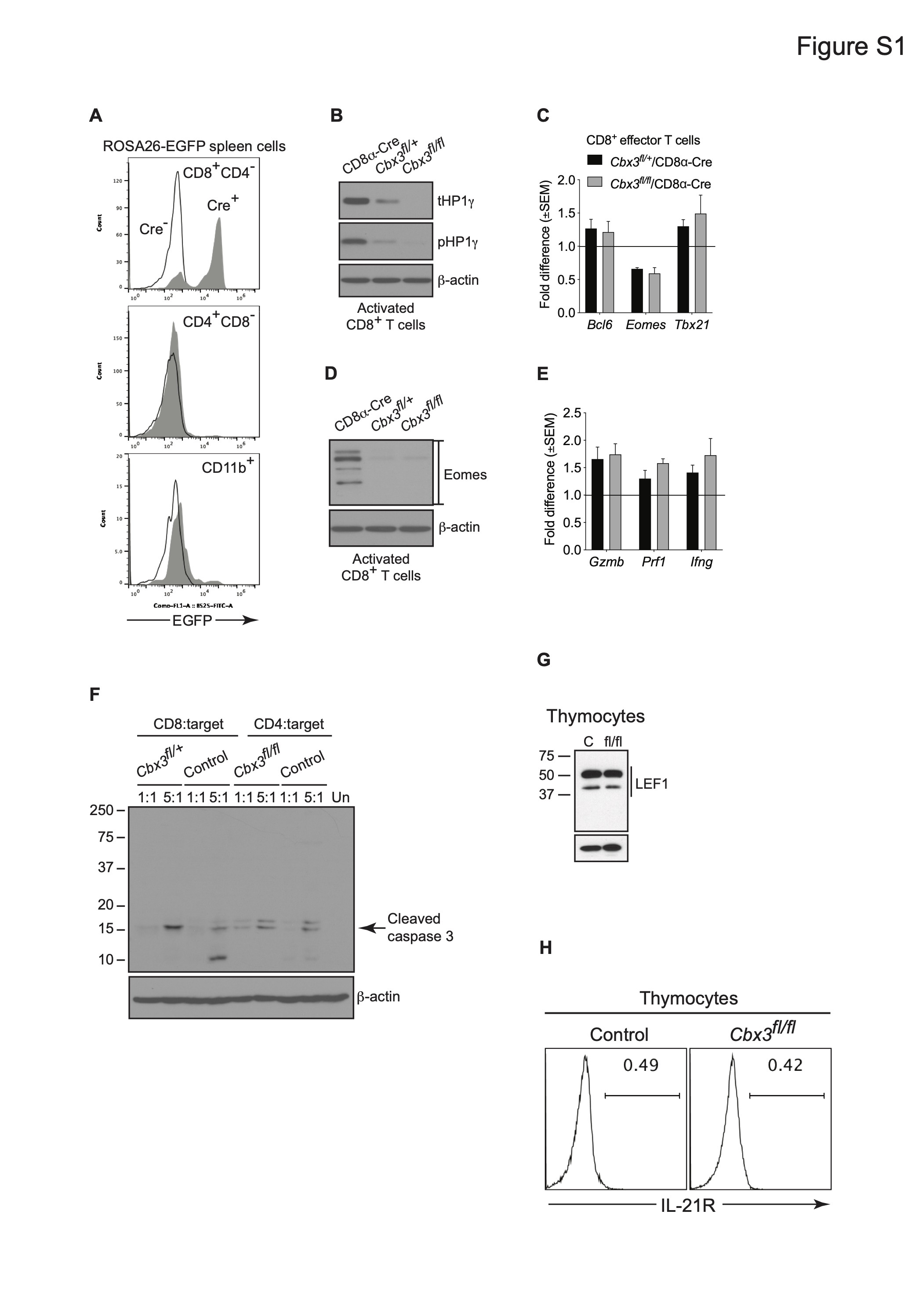

Supplement: Supplementary Figure 1 — Enhanced effector functions of Cbx3/HP1γ-deficient CD8+ T cells. (A) Cre activity (EGFP+) was restricted to CD8+CD4– spleen T cells from a cross between the reporter mouse ROSA-EGFP and the CD8α-Cre mouse. (B) Western immunoblot of total (tHP1γ) and phosphorylated (pHP1γ) Cbx3/HP1γ levels were evaluated in day 5 activated/differentiated pooled spleen and peripheral lymph node (pLN) CD8+ T cells using plate-bound anti-CD3/CD28 + IL-2 (10 IU/ml). Cbx3fl/+ and Cbx3fl/fl: CD8+ T-cell-restricted deletion of Cbx3/HP1γ in mice using the CD8α-Cre strain; collectively designated as Cbx3/HP1γ-deficient; representative of 3 experiments; n = 3 mice. (C) Relative expression of Bcl6, Eomes and Tbx21 was quantified by RT-qPCR using RNA samples of day 5 activated/differentiated CD8+ T cells. Unit 1 indicates no change; n = 3; representative of 3 experiments. (D) Western immunoblot showed EOMES expression in same cells as (B). (E) Relative expression of Gzmb, Prf1 and Ifng was etermined by RT-qPCR using same RNA samples as in (C). (F) Cleaved (activated) caspase 3 (CC3) in protein lysate of washed cells was detected 24 hours after coculture using an antibody specific to CC3. CD8:target = effector CD8+ T cells cocultured with target; control: CD8α-Cre; Cbx3fl/+: CD8-restricted deletion of Cbx3/HP1γ with the CD8α-Cre mouse. CD4:target = day 2 activated CD4+ T cells from control (CD4-Cre) or Cbx3fl/fl mice cocultured with target; Cbx3fl/fl: CD4-restricted deletion of Cbx3/HP1γ with the CD4-Cre mouse; target: B16 melanoma tumor cells. 1:1= 1 effector cell to 1 target cell; 5:1 = 5 effector cells to one target cell; Un: no added effector cells; representative of 3 experiments. (G) Western immunoblot showed LEF-1 expression in thymocytes from control and Cbx3/HP1γ-deficient mice; representative of 3 experiments. (H) Flow histograms showed lack of IL-21R expression on thymocytes from control and Cbx3/HP1γdeficient mice; representative of 3 experiments. [file Image_1.jpeg]

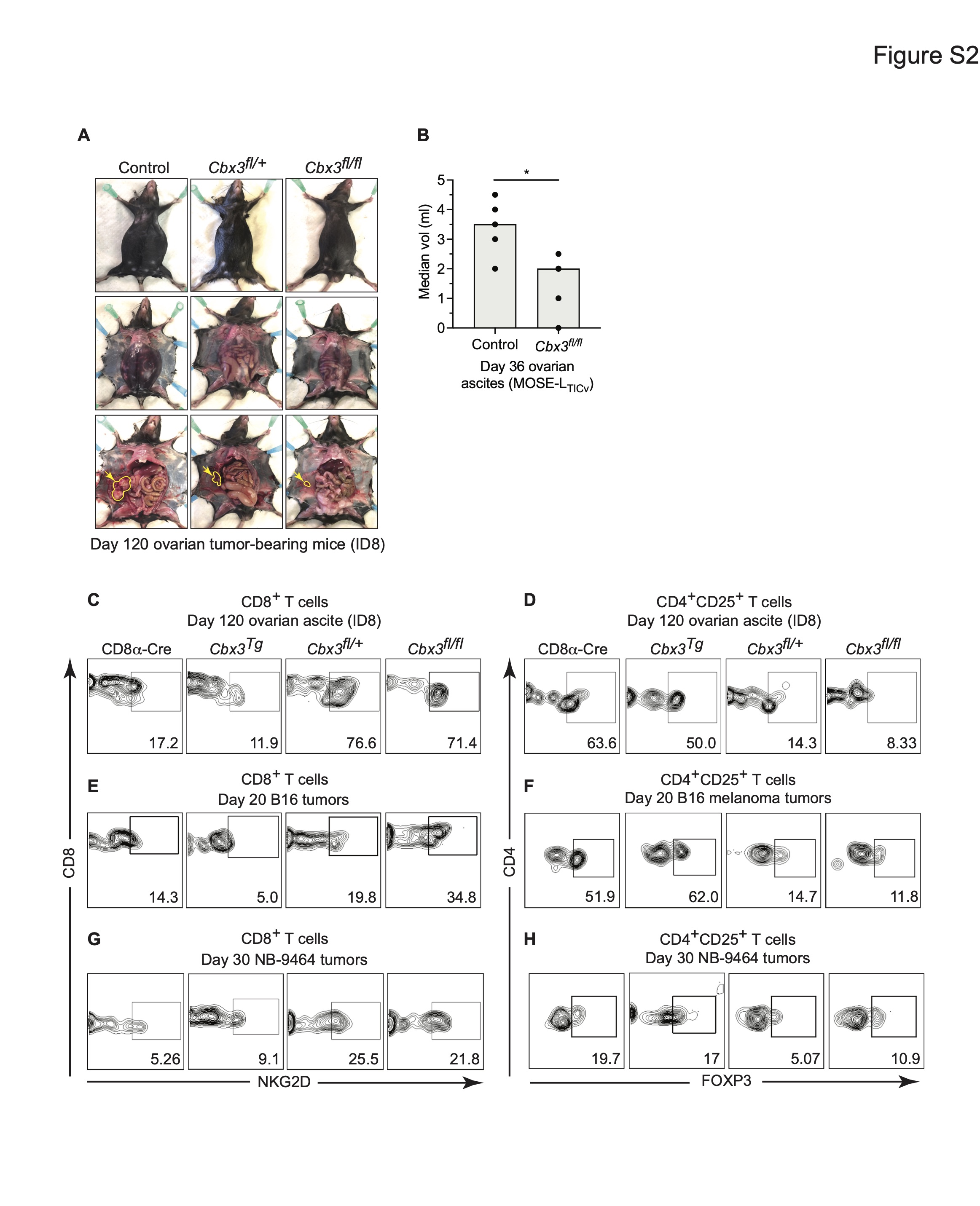

Supplement: Supplementary Figure 2 — Reduction of ovarian ascites in Cbx3/HP1γ-deficient mice. (A) Images showed ovarian ascites in control and Cbx3/HP1γ-deficient mice injected (IP) with the syngeneic mouse ID8 cell line; control: CD8α-Cre; Cbx3fl/+ and Cbx3fl/fl: Cbx3/HP1γ-deficient mice; yellow outlines: tumor nodules (supporting data for Figures 3A, B ). (B) Ovarian ascites in control and Cbx3/HP1γ-deficient mice injected (IP) with the syngeneic mouse MOSE-LTICv cell line were quantified and graphed as group median; control: CD8α-Cre; Cbx3fl/fl: Cbx3/HP1γ-deficient mice; Graphpad student unpaired t-test; *p≤0.05; n = 4-5.; representative of 2 experiments. (C, E, G) Flow analysis showed frequencies of CD8+NKG2D+ effector T cells in ovarian ascites, B16 and NBL tumors from control (CD8α-Cre or wt), Cbx3Tg, Cbx3fl/+ and Cbx3fl/fl mice; Cbx3Tg: T-cell-restricted expression of Cbx3/HP1γ driven by the human Cd2 promoter (supporting data for Figures 3H, J, L ). (D, F, H) Flow analysis depicted the frequencies of CD4+FOXP3+ regulatory T cells (Tregs) in ovarian ascites, B16 and NBL tumors from control (CD8α-Cre or wt), Cbx3Tg, Cbx3fl/+ and Cbx3fl/fl mice (supporting data for Figures 3I, K, M ). [file Image_2.jpeg]

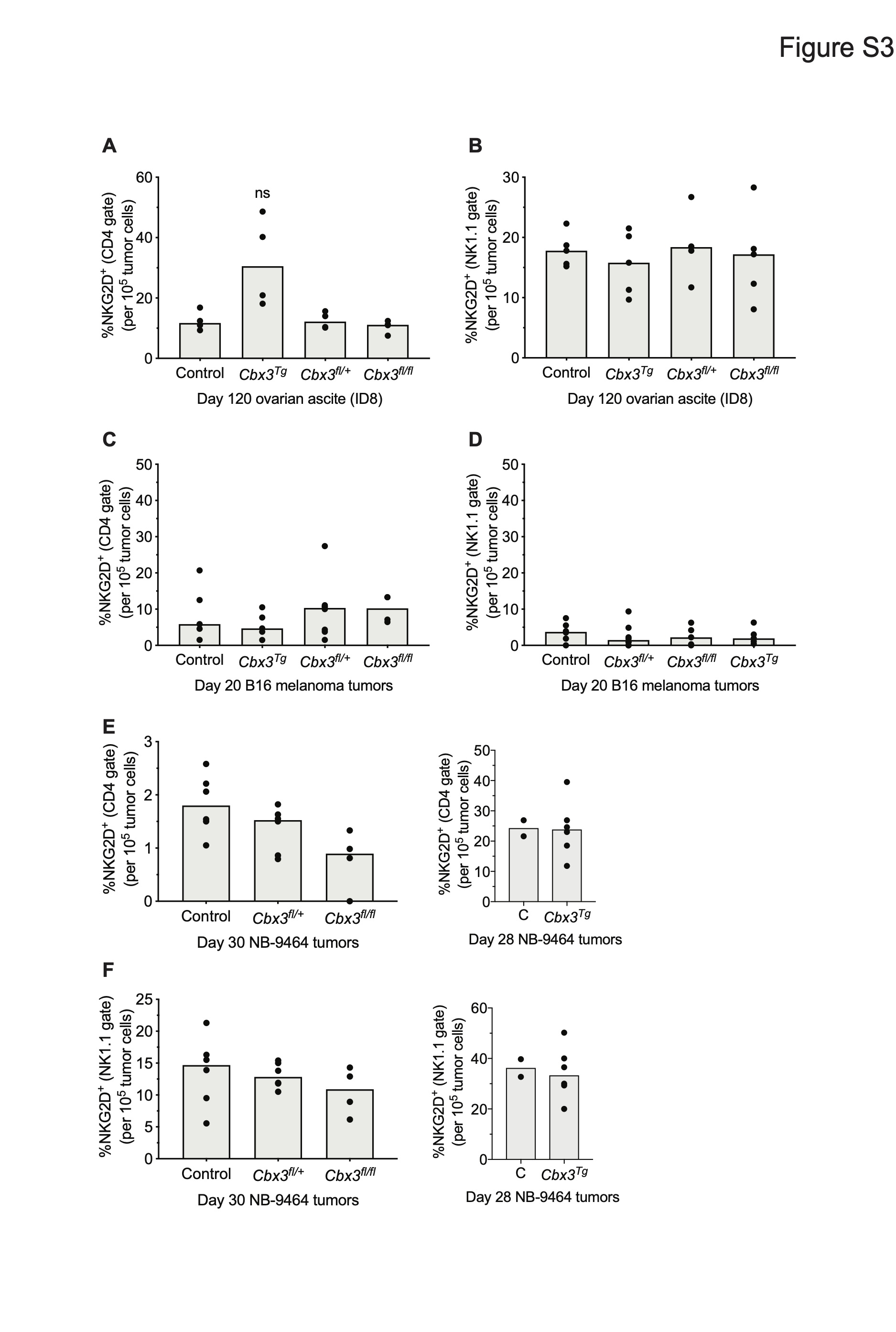

Supplement: Supplementary Figure 3 — No enrichment of CD4+NKG2D+ or NK1.1+NKG2D+ T cells in tumors (supporting data for Figure 3). (A, C, E) Frequencies of CD4+NKG2D+ T cells in ovarian ascites, B16 and NBL tumors were extracted from flow analysis; bars: group median; Graphpad unpaired student t-test: ns, not significant; each symbol = one mouse; n = 2-8. (B, D, F) Frequencies of NK1.1+NKG2D+ T cells in ovarian ascites, B16 and NBL tumors were extracted from flow analysis; bars: group median; each symbol = one mouse; n = 2-8. [file Image_3.jpeg]

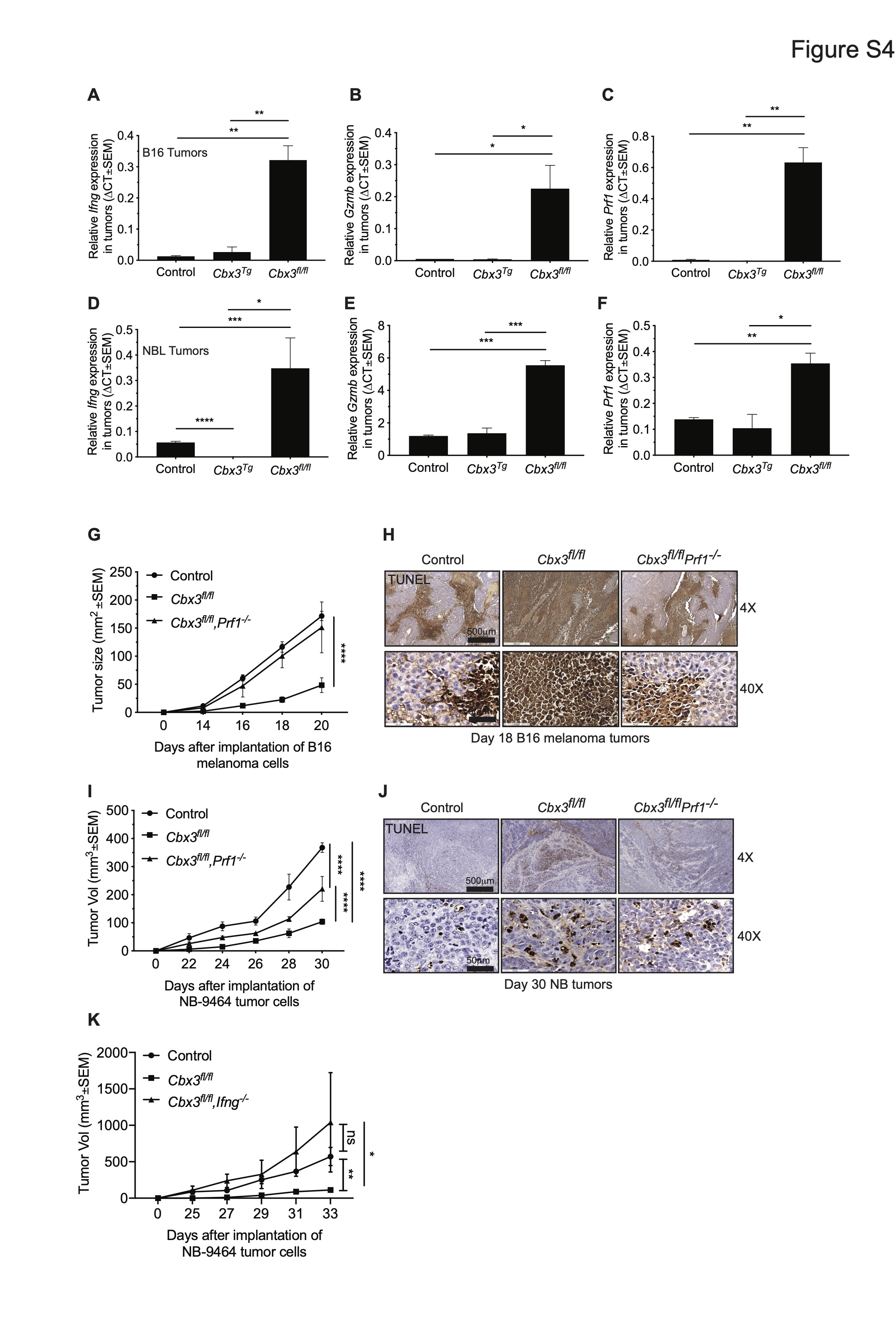

Supplement: Supplementary Figure 4 — Prf1, Gzmb and Ifng expression in tumors. (A–C) Relative expression of Ifng, Gzmb and Prf1 in B16 melanoma tumors were quantified by RT qPCR and normalized to Gapdh; Graphpad unpaired student t-test: *p≤0.05, **p≤0.01, n = 4 tumors from each mouse strain; representative of 4 experiments. (D–F) Relative expression of Ifng, Gzmb and Prf1 in NBL tumors was normalized to Gapdh; Graphpad unpaired student t-test: *p≤0.05, **p≤0.01, ***p≤0.001; n = 3 tumors from each mouse strain; representative of 3 experiments. (G) B16 melanoma tumor growth in Cbx3fl/fl,Prf1-/- (Cbx3/HP1γ-deficient mice lacking Prf1), Cbx3fl/fl (Cbx3/HP1γ-deficient) and control (CD8α-Cre and wt) mice was measured; Graphpad two-way ANOVA; ****p≤0.0001;n = 6; representative of 2 experiments. (H) Apoptotic cells (brown) in B16 melanoma tumors from mice in (A) were identified by TUNEL staining; representative of 2 experiments. (I) NBL tumor burden in mice implanted with NB-9464 tumor cells was measured; Cbx3fl/fl,Prf1-/-: Cbx3/HP1γ-deficient mice lacking Prf1; Cbx3fl/fl: Cbx3/HP1γ-deficient; control: CD8α-Cre and wt; Graphpad two-way ANOVA; ****p≤0.0001; n = 3-5; representative of 2 experiments. (J) Apoptotic cells (brown) in NBL tumors from mice in (C) were identified by TUNEL staining; representative of 2 experiments. (K) NBL tumor growth in Cbx3fl/fl,Ifng-/- (Cbx3/HP1γ-deficient mice lacking Ifng) was evaluated; Cbx3fl/fl (Cbx3/HP1γ-deficient) and control (CD8α-Cre and wt) mice; Graphpad two-way ANOVA; *p≤0.05, **p≤0.01; n = 3-4; representative of 2 experiments. [file Image_4.jpeg]

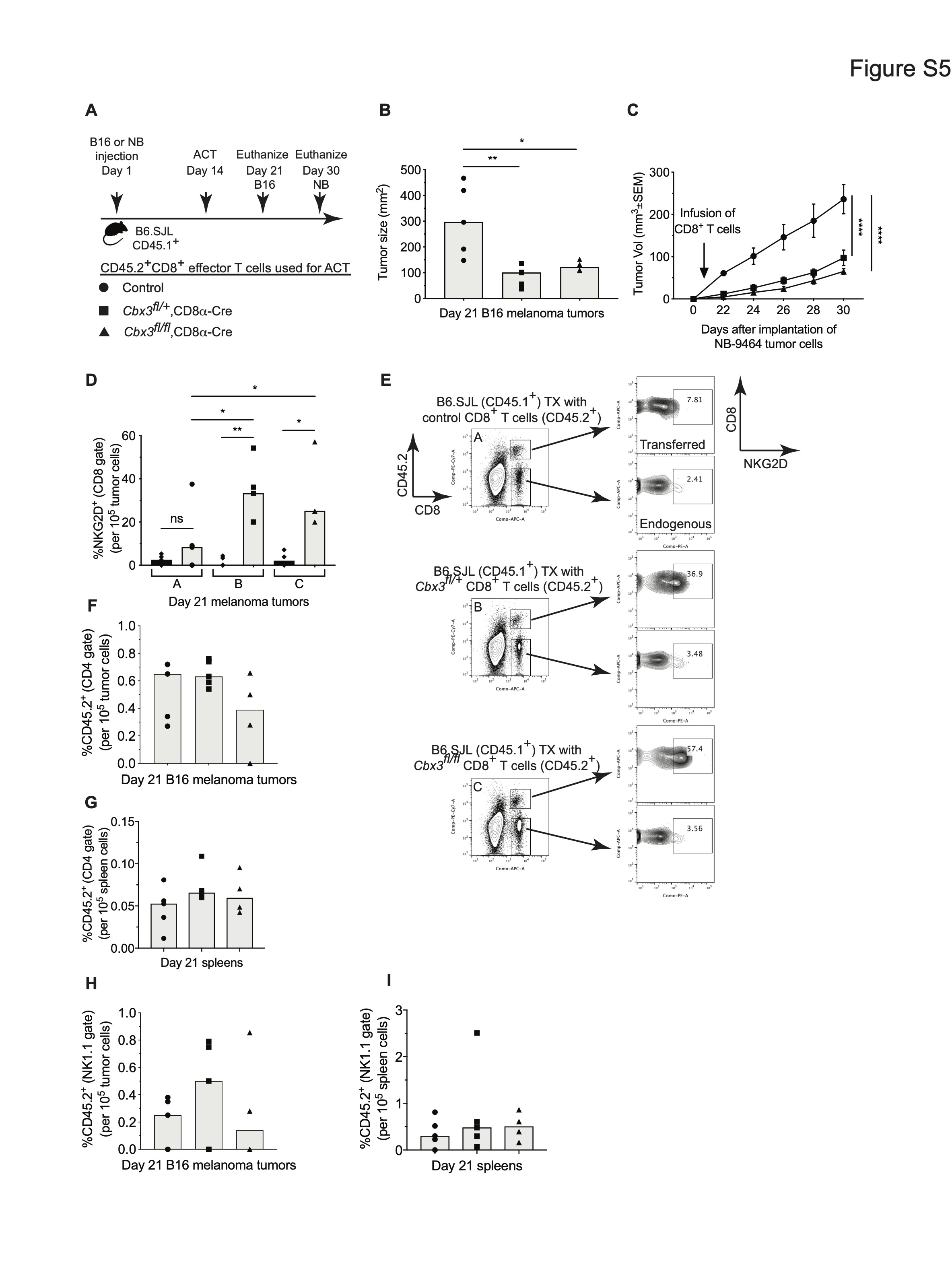

Supplement: Supplementary Figure 5 — Direct cause of tumor rejection by Cbx3/HP1γ-deficient CD8+ effector T cells. (A) Schematic of ACT. (B) 10 days after implantation of B16 tumor cells, B6.SJL recipients (CD45.1+) were treated once with exogenous CD8+ effector T cells (CD45.2+); bars: group median; Graphpad unpaired student t-test: *p≤0.05, **p≤0.01; each symbol = one mouse; n = 4-5 recipients; representative of 2 experiments. (C) 14 days after implantation of N-9464 tumor cells, B6.SJL recipients were treated once with exogenous CD8+ effector T cells; Graphpad two-way ANOVA: ****p≤0.0001; n = 5 recipients; representative of 2 experiments. (D) Frequencies of CD8+ effector T cells in B16 melanoma tumors after ACT; A: B6.SJL mice treated with control CD8+ T cells, B: B6.SJL mice treated with Cbx3fl/+ CD8+ T cells, C: B6.SJL mice treated with Cbx3fl/fl CD8+ T cells; grey bars: transferred CD8+ effector T cells (CD45.2+); black bars: endogenous CD8+ effector T cells (CD45.1+); bars: group median; Graphpad unpaired student t-test: *p≤0.05, **p≤0.01, ns: not significant; each symbol = one mouse; n = 4-5 recipients from B. (E) Staining and gating strategies for panel D; gated on total live tumor cells obtained from tumors of B6.SJL recipients from B. (F, G) Frequencies of CD4+CD45.2+ T cells in B16 melanoma tumors (F) and spleens (G) from B6.SJL recipients treated with control (CD8α-Cre) or Cbx3/HP1γ-deficient CD8+ effector T cells; data were extracted from flow analysis; bars: group median; each symbol = one mouse; n = 4-5 recipients as in (D). (H, I) Frequencies of NK1.1+CD45.2+ T cells in B16 tumors (H) and spleens (I) from B6.SJL recipients treated with control or Cbx3/HP1γ-deficient CD8+ effector T cells; data were extracted from flow analysis; bars: group median; each symbol = one mouse; n = 4-5 recipients from B. [file Image_5.jpeg]

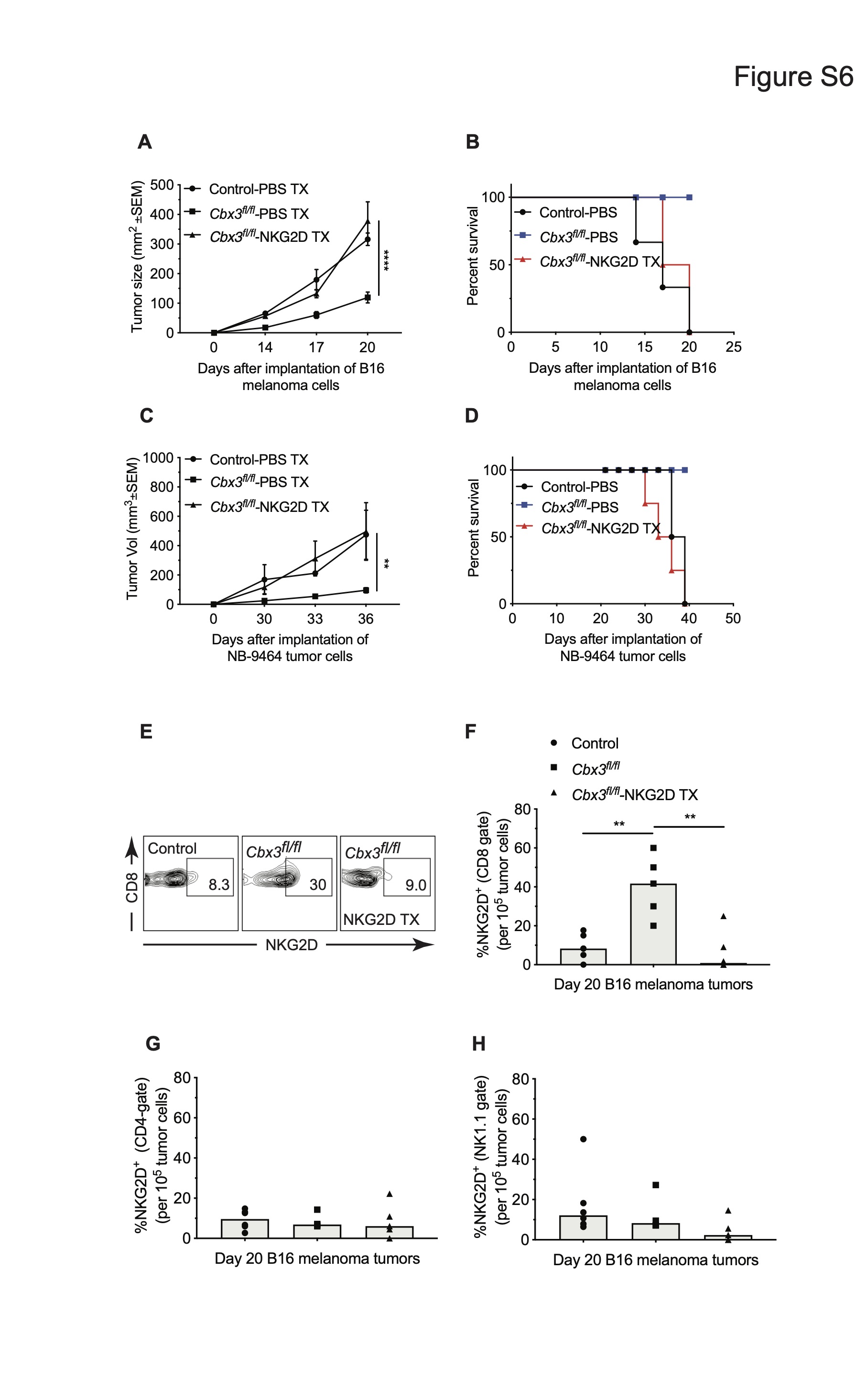

Supplement: Supplementary Figure 6 — Effects of NKG2D blockade on tumor growth. (A) B16 melanoma tumor burden was assessed after NKG2D blockade using the anti-mouse NKG2D antibody HMG2D; Graphpad two-way ANOVA: ****p≤0.0001; Cbx3fl/fl-NKG2D TX: Cbx3fl/fl mice injected with HMG2D one day after injection of tumor cells; control and Cbx3fl/fl mice were injected with PBS; n = 5 received Ab, 10 received PBS; representative of 2 experiments. (B) Survival curves of B16 tumor-bearing mice after NKG2D blockade. (C) NBL tumor burden was evaluated after NKG2D blockade; Graphpad two-way ANOVA: **p≤0.01; n = 4 received Ab, 10 received PBS; representative of 2 experiments. (D) Survival curves of NBL tumor-bearing mice after NKG2D blockade. (E, F) Flow analysis showed frequencies of CD8+NKG2D+ T cells in B16 melanoma tumors; bars: group median; Graphpad student unpaired t-test: **p≤0.01; each symbol = one mouse; Cbx3fl/fl-NKG2D TX: Cbx3/HP1γ-deficient mice receiving the anti-mouse NKG2D antibody HMG2D; control and Cbx3/HP1γfl/fl: PBS treated. (G, H) Frequencies of CD4+NKG2D+ and NK1.1+NKG2D+ T cells in B16 melanoma tumors in (A). [file Image_6.jpeg]

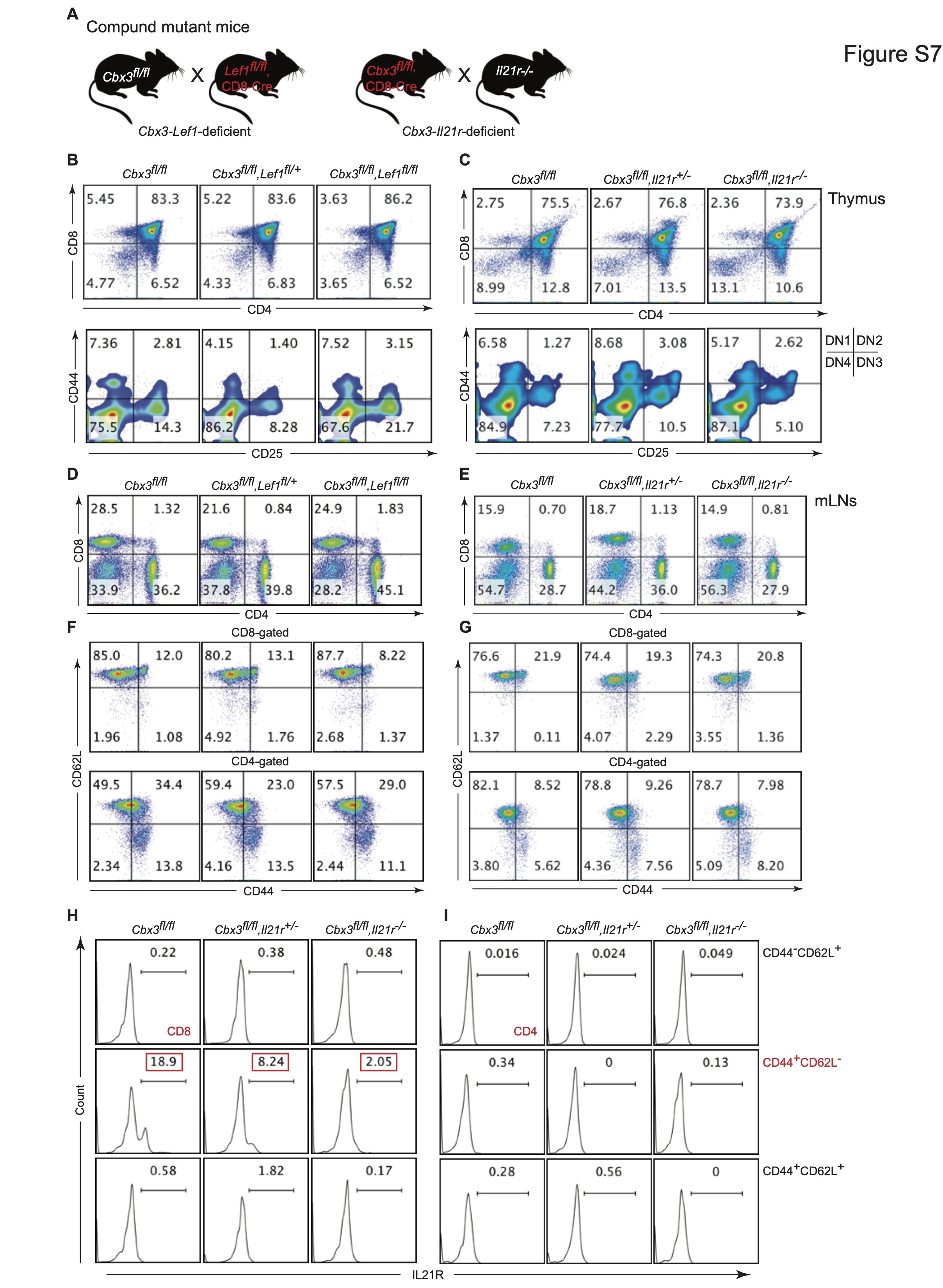

Supplement: Supplementary Figure 7 — Normal T-cell development in the thymus of compound mutant mice. (A) Generation of compound mutant mice. (B, C) T-cell development in the thymus of Cbx3-Lef1-deficient and Cbx3-Il21r-deficient mice was assessed; numbers: percent cells; DN1-DN4: double negative (CD4–CD8–) progenitor T-cell populations gated from bottom left quadrants of top flow plots. (D, E) CD4+ and CD8+ ratio was evaluated in mLNs from compound mutant mice; numbers: percent cells. (F, G) mLN naïve (CD44–CD62L+), effector (CD44+CD62L–) and memory (CD44+CD62L+) cells in CD4+ as well as CD8+ T-cell compartments of compound mutant mice were assessed; numbers: percent cells. (H, I) Flow analysis was done to evaluate IL21R expression on mLN CD8+ (H) and CD4+ (I) effector (CD44+CD62L–), naïve (CD44–CD62L+) and memory (CD44+CD62L+) T cells from Cbx3/HP1γ-deficient and Cbx3-Il21r-deficient mice; numbers: percent cells. For all panels, n = 3 for each genotype; representative of 3 experiments. [file Image_7.jpeg]

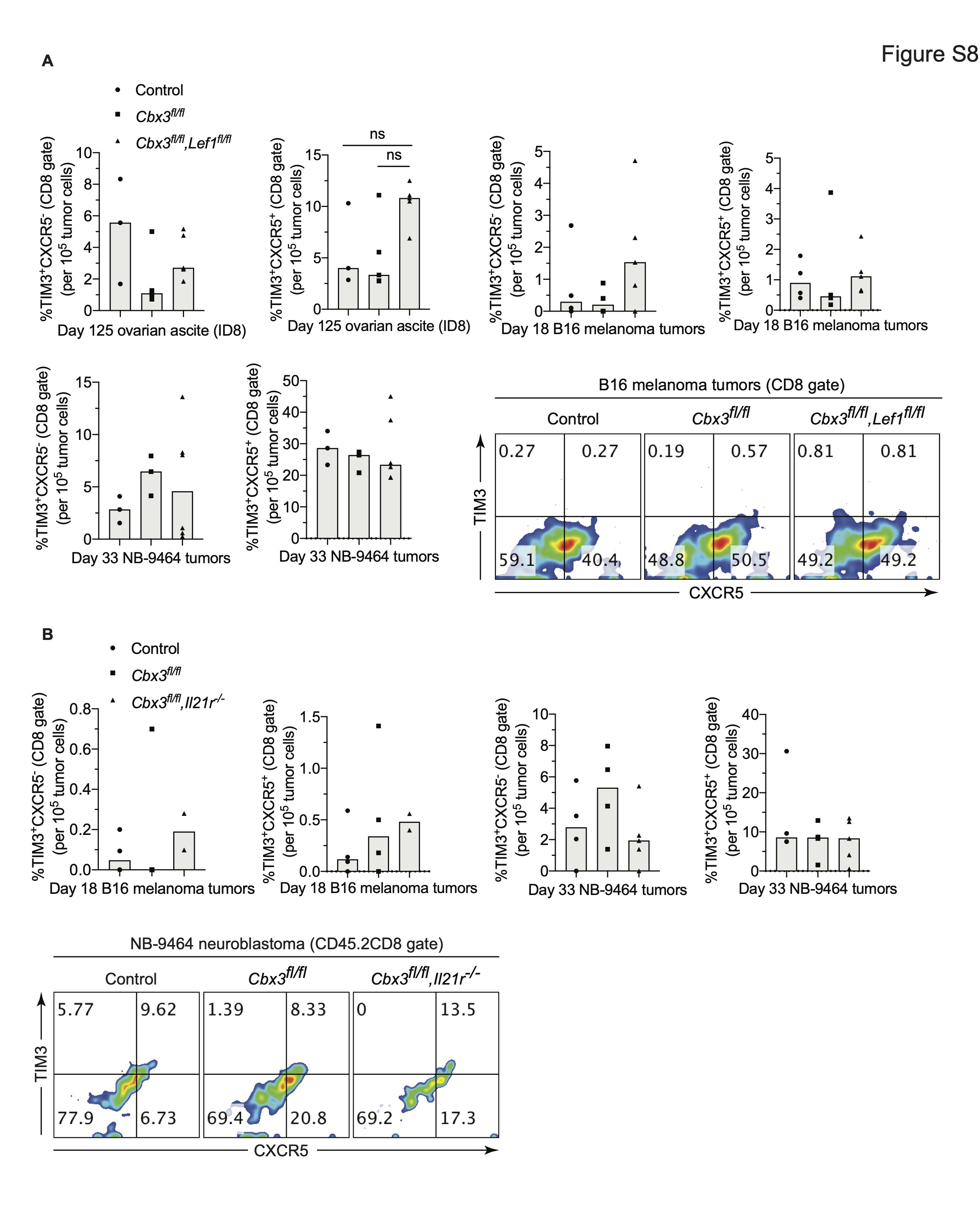

Supplement: Supplementary Figure 8 — Analysis of CD8+ progenitor and terminally exhausted T cells. (A) Frequencies of progenitor exhausted (TIM3+CXCR5+) and terminally exhausted (TIM3+CXCR5–) CD8+ T cells in all tumors from Cbx3fl/flLef1fl/fl (Cbx3-Lef1-deficient), Cbx3fl/fl (Cbx3/HP1γ-deficient) and control (CD8α-Cre or wt) mice were assessed; n = 3-5; representative of 2 experiments. (B) Progenitor exhausted (TIM3+CXCR5+) and terminally exhausted (TIM3+CXCR5–) CD8+ T cells were identified in tumors from B6.SJL mice treated with Cbx3fl/flIl21r-/-, Cbx3fl/fl or control CD8+ effector T cells (CD45.2+); n = 3-5 recipients; representative of 2 experiments. [file Image_8.jpeg]

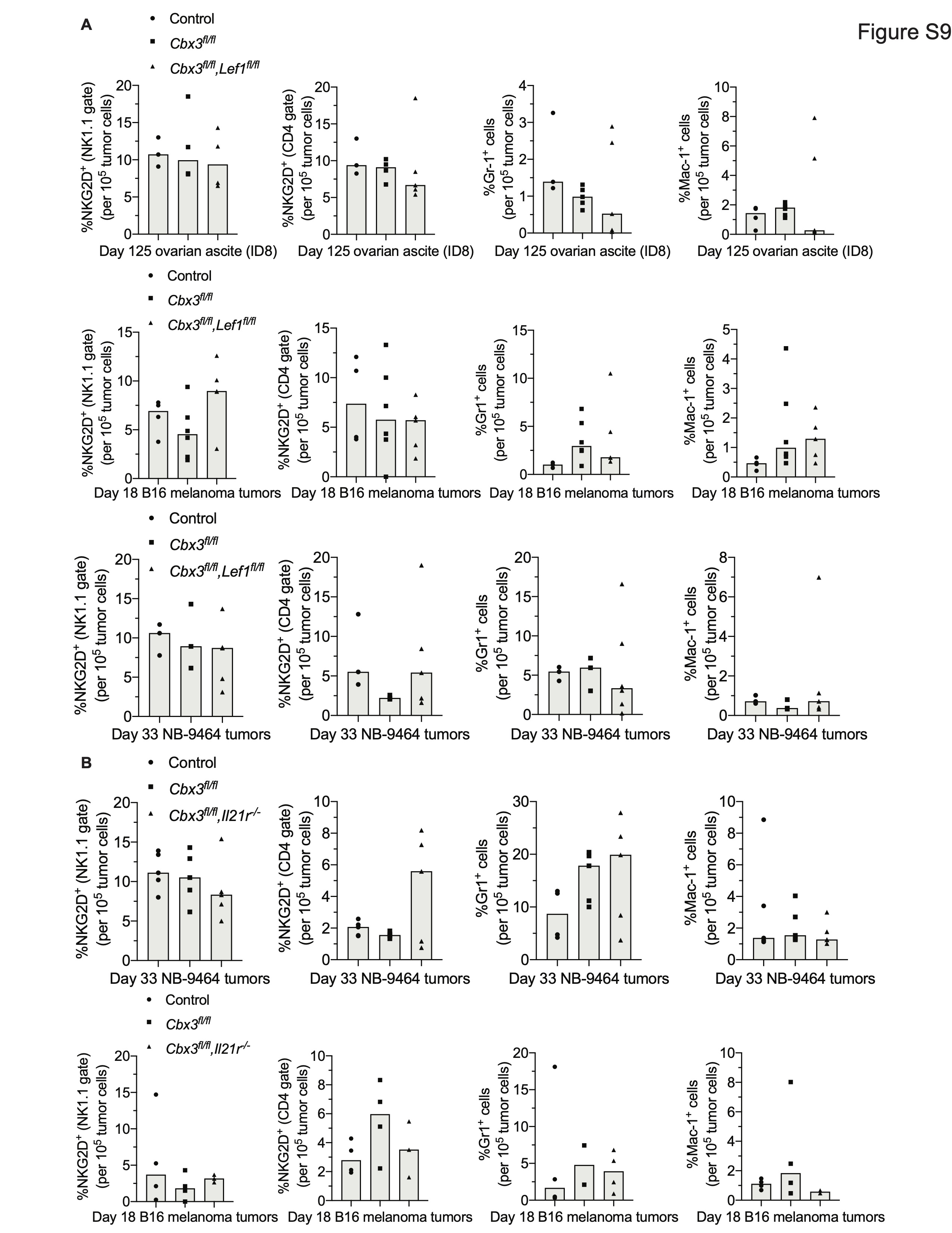

Supplement: Supplementary Figure 9 — Analysis of tumor infiltrating immune populations. (A) Frequencies of NK1.1+NKG2D+, CD4+NKG2D+ T, Gr1+ and Mac1+ myeloid cells in tumors from Cbx3fl/flLef1fl/fl (Cbx3-Lef1-deficient) and control animals; representative of 2 experiments. (B) Frequencies of endogenous NK1.1+NKG2D+, CD4+NKG2D+ T, Gr1+ and Mac1+ myeloid cells in tumors from B6.SJL mice treated with Cbx3fl/flIl21r-/- and control CD8+ effector T cells; representative of 2 experiments. [file Image_9.jpeg]
