# Supplementary material for: Targeting Cbx3/HP1γ Induces LEF-1 and IL-21R to Promote Tumor-Infiltrating CD8 T-Cell Persistence
Source: Front Immunol. 2021 Oct 6;12:738958. doi: 10.3389/fimmu.2021.738958 (PMC8549513; doi:10.3389/fimmu.2021.738958)
Supplement: Supplementary file 10 [file Table_1.pdf]

Table S1. ChIP-Seq analysis. Regions within *Lef1* and *Il21r* loci bound by *Cbx3*/HP1γ in wild type mouse CD8+ T cells activated/differentiated for 5 days with plate-bound anti-CD3/CD28 + 10 IU hrIL-2

Chr	Start	End	Gene List	Dist to Start	Position
3	131,105,000	131,108,199	*Lef1*	-3,872	upstream
3	131,109,000	131,127,399	*Lef1*	7,728	in gene
3	131,132,200	131,135,399	*Lef1*	23,328	in gene
3	131,136,400	131,140,599	*Lef1*	28,028	in gene
3	131,154,600	131,163,199	*Lef1*	48,428	in gene
3	131,174,400	131,180,399	*Lef1*	66,928	in gene
3	131,205,800	131,209,199	*Lef1*	97,028	in gene
3	131,214,600	131,216,999	*Lef1*	105,328	in gene
7	125,567,200	125,611,599	*Il21r*	-14030	upstream
7	125,616,200	125,624,399	*Il21r*	16,870	in gene
7	125,625,200	125,641,399	*Il21r*	29870	in gene

Upstream: upstream of transcription start site (TSS)
