# Supplementary material for: Targeting Cbx3/HP1γ Induces LEF-1 and IL-21R to Promote Tumor-Infiltrating CD8 T-Cell Persistence
Source: Front Immunol. 2021 Oct 6;12:738958. doi: 10.3389/fimmu.2021.738958 (PMC8549513; doi:10.3389/fimmu.2021.738958)
Supplement: Supplementary file 11 [file Table_2.pdf]

Table S2. Primer sequences for ChIP-qPCR

Genes	Forward	Reverse
Lef1		
-2	GACCAACCACGTCCTATGCAG	GTCAGCATTTAAAACTTTGC
-1	GTCGCCCTTAAAAGTCCTGTC	AGGCAGGCAGCACTGGGCAG
TSS	CTAATTCTCGCCTTATTCCCTC	GTCCTTTGCCAGTCTTTCTTC
+1	CTCATCATCACAAACTTTATTC	CGGGCTGCAGACGTCCGCGTTAG
+2	CTGGCAGCCTAGCCTAGTGC	GCAGGAGCCTCTGCGATTGC
+3	GTGCGCACGTGTGTGAGCAG	TTTTAATGCACCGCGTCCCTC
+4	GTTGGGATCAGGCCCTGGATC	GTGTCTCTATTTACTCTACTC
+5	CTCTGGTGGTTTTTTATTTTTG	CAGTCTCCTAAGCATGGCTT
+6	CCCCTGATCGCCTAGTATATTTAG	CTGACATTTCAAAATGGTGC
+7	AGAAGCCTTTCATTTTTAACC	GAGGCTGCCTTCCTTCCGG
+8	GGAGATGCGCAAGCCTGC	GGCTTTGGTGTTTGTTGTTG
+9	CACACACACACCGTTCTCAA	CCTCAGCCACCAACCCTTG
+10	CACCGTCGCAGCCATTTCTC	GGTTTTTGAAATTAACTGCG
+11	CCAGTGTCAGTGAGATTTAG	GAAAGGAGAATCTGGAAGG
+12	GTTTCCCAGAGAAGCCCGTG	GTTCAGATTGGGAGAGGGAC
Il21r		
-4	CAGGAGGAGGGGCAAGTGCA	AGTCTTGGTTATCACACCAG
-3	AGAGTTGAGCAGAAAGACAC	ATTAATGACATGAGAGGGCA
-2	TTTGAAGACCCAGTAGGAGG	TTGCTCTGGCTCCAGACACA
-1	GTCAAGAACATGCCACCCTGC	TGGGCTGACTAGTTTGGGGC
TSS	AATCACTCTCCACCTTACGT	AGAAACCCCACCCCCATGGC
+1	CAGTAACGCCTCATTTCTCC	AACTCCTGTCATATGTTGCA
+2	ACTTACTTGGAATGTGAAGG	GATGGCTATAAATGTCTCCT
+3	GGAGACTTCAGTTGTGACTT	TCTCAATGGCACGCAGAGCG
+4	ACAGTGGAGCCCAGAGCAGC	GGATGTGCTAACACACAACC
