# Supplementary material for: Targeting Cbx3/HP1γ Induces LEF-1 and IL-21R to Promote Tumor-Infiltrating CD8 T-Cell Persistence
Source: Front Immunol. 2021 Oct 6;12:738958. doi: 10.3389/fimmu.2021.738958 (PMC8549513; doi:10.3389/fimmu.2021.738958)
Supplement: Supplementary file 12 [file Table_3.pdf]

Table S3. Primer sequences for RT-qPCR

Genes	Forward	Reverse
Gzmb	ACTGCTGCTCACTGTGAAGG	CACAGCTCTAGTCCTCTTGG
Prf1	TCCAAGGTAGCCAATTTTGC	GAGGAGATGAGCCTGTGGTA
Ifng	GCACAGTCATTGAAAGCCTA	GAAAGAGATAATCTGGCTCT
Il21r	GCAGCTTTGTCCTGGCTGAG	CACAGCATAGGGGTCTCTGAG
Ccl2	CCGGCTGGAGCATCCACGTGTTG	GACACCTGCTGCTGGTGATCCTC
Ccr2	ATGGCGCAAGGCTATTTG	GGATACTTCGTGTAAATAGAG
Cxcl9	CCCTAGTGATAAGGAATGCAC	GGATCTAGGCAGGTTTGATC
Cxcl10	CCCAAGTGCTGCCGTCATTTTC	CAGGATAGGCTCGCAGGGATG
Cxcr3	CCTACGATTATGGGGAAAAC	CTCAGTAGCACAGCAGCCAC
Ccl28	GCTCACACTCATGGCTGTGGCTG	CTGCATGAACTCACTCTTTCCAG
Ccr10	GCAGGTCTCCTGGGGACTTTAC	CAGGGAGACACTGGGTTGGAAGG
Cxcl12	ATCGCCAGAGCCAACGTCAAG	CGGTCCATCGGCAGGAAGCG
Cxcr4	CGGACAAGTACCGGCTGCAC	CTGCTGTAGAGGTTGACAGTG
